# From 2,5-Diformyl-1,4-dihydropyrrolo[3,2-*b*]pyrroles to Quadrupolar, Centrosymmetric Two-Photon-Absorbing A–D–A Dyes

**DOI:** 10.1021/acs.orglett.2c00718

**Published:** 2022-03-28

**Authors:** Paweł Kowalczyk, Mariusz Tasior, Shuhei Ozaki, Kenji Kamada, Daniel T. Gryko

**Affiliations:** † Institute of Organic Chemistry, Polish Academy of Sciences, Kasprzaka 44/52, 01-224 Warsaw, Poland; ‡ NMRI, National Institute of Advanced Industrial Science and Technology (AIST), Ikeda, Osaka 563-8577, Japan; § Department of Chemistry, Graduate School of Science and Technology, Kwansei Gakuin University, Sanda 669-1337, Japan

## Abstract

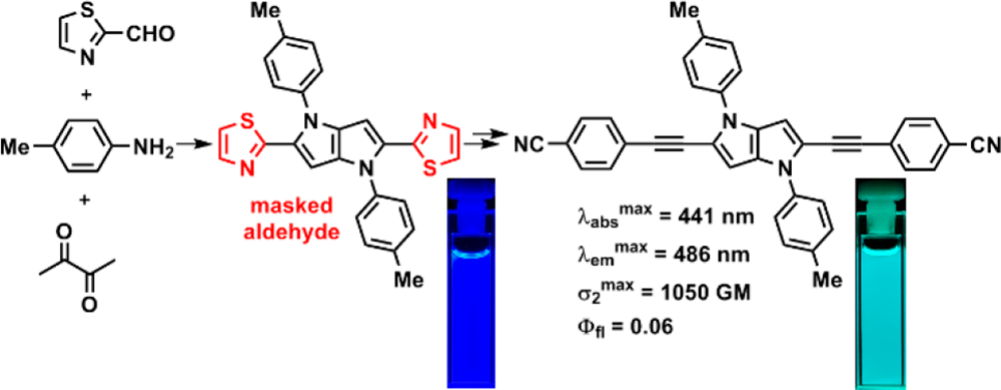

An original approach has been developed for the insertion of formyl substituents at positions 2 and 5 of 1,4-dihydropyrrolo[3,2-*b*]pyrroles by conversion of thiazol-2-yl substituents. The synthetic utility of these formyl groups was investigated, and a series of centrosymmetric A−π–D−π–A frameworks were constructed. The two-photon absorption of the quadrupolar pyrrolo[3,2-*b*]pyrrole possessing two dicyanovinylidene flanking groups is attributed to an S_0_ → (S_1_) → S_4_ transition which has a large TPA cross-section (1300 GM) for a molecule of this size.

1,2,4,5-Tetraaryl-1,4-dihydropyrrolo[3,2-*b*]pyrroles^1,2^ (TAPPs) have attracted an increasing amount of attention since the discovery of a multicomponent reaction affording their synthesis in a one-pot process.^3^ Although this reaction has been heavily optimized and its scope enlarged to include substrates of varying electronic character, or are sterically hindered and/or heterocyclic, so far only aromatic aldehydes and aromatic amines have proved to be suitable starting materials.^4,5^ To date, all attempts to use cinnamaldehydes or arylpropargylaldehydes have failed, limiting the possibilities of structural modification.

Although the dihedral angles between the four aromatic substituents and the heterocyclic core are in the range 35–50°, TAPPs decorated with electron-withdrawing groups at the 2- and 5-positions exhibit exceptionally strong electronic coupling, leading in turn to reasonable two-photon absorption (TPA) cross sections,^6−8^ strong solvatofluorochromism^8^ and excited-state symmetry breaking.^8,9^ Given the potential applications of TAPPs in the areas of organic light-emitting diodes,^10^ resistive memory devices,^11^ bulk heterojunction organic solar cells,^12^ dye-sensitized solar cells,^13^ aggregation-induced emission,^14^ MOFs,^15^ direct solvent probing via H-bonding interactions,^16^ and photochromic analysis of halocarbons,^17^ together with synthesis of PAHs heteroanalogues,^18^ it would be of general benefit to find a methodology that allows the synthesis of pyrrolo[3,2-*b*]pyrroles bearing substituents other than aromatic. In this paper, we present the first solution to this problem.

Our approach to this started with exploration of the methodology developed by Altmann and Richheimer^19^ and then further refined by Corey and Boger^20^ in the 1970s, which utilized thiazoles and benzothiazoles as carbonyl equivalents.^21^ This transformation proceeds via quaternization of the azole nitrogen atom followed by reduction and finally cleavage of the *N*,*S*-acetals with mercury or silver salts. Given that recent synthetic breakthroughs have enabled the preparation of 2,5-bis(thiazol-2-yl)pyrrolo[3,2-*b*]pyrroles in reasonable quantities, and the fact that TAPPs are in general compatible with alkylating/reducing agents, we came to the conclusion that the above reaction sequence could provide access to formyl-substituted pyrrolo[3,2-*b*]pyrroles, which in turn could open the door for further derivatization via Knoevenagel condensation, Wittig reaction, etc. To test this hypothesis, we synthesized TAPP **4** from 2-formylthiazole (**1**) (Scheme 1). Its quaternization with methyl triflate proceeded quantitatively. In addition, the reduction to double *N*,*S*-acetal using NaBH_4_ did not pose any major problems. However, hydrolysis using copper(II) chloride or mercury(II) chloride gave 5% and 28% yields, respectively. Because of the low yield of the former and toxicity of the latter, we decided to test silver nitrate. A short optimization of the conditions led to cleavage in an acceptable 41% yield.

**Scheme 1:**
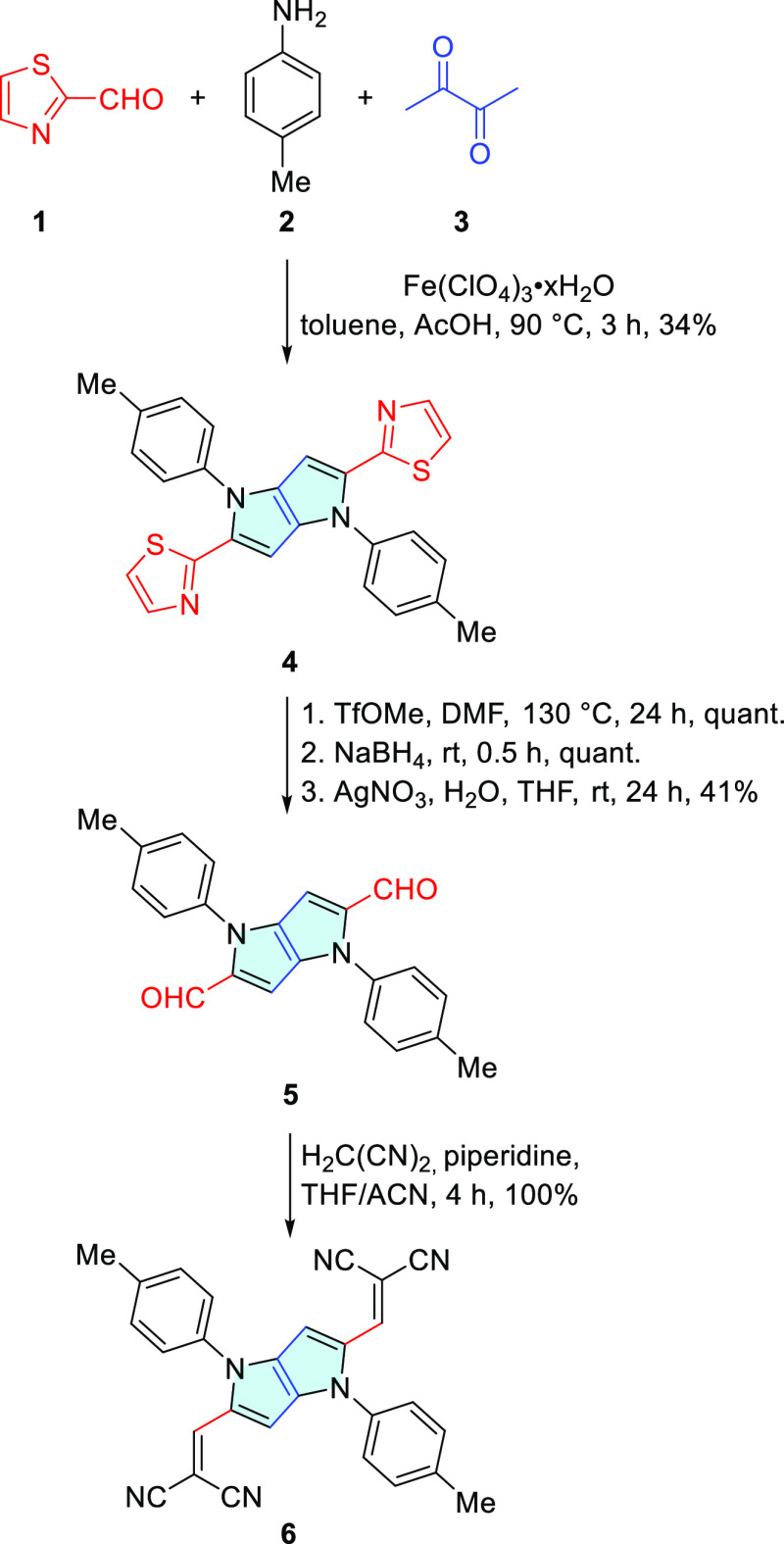
Synthesis of Aldehyde **5** and Dye **6**

With the crucial dialdehyde **5** in hand, we attempted Knoevenagel condensation with malononitrile, which afforded the expected product **6** in high yield (Scheme 1). Unfortunately, condensation with other nucleophiles did not proceed as smoothly, apparently due to the low reactivity of the formyl groups. This was anticipated, as the pyrrolo[3,2-*b*]pyrrole core is extremely electron rich, and while the reaction with the first formyl group was relatively efficient due to the electron-withdrawing nature of the neighboring formyl group, the subsequent reaction of the second formyl group was much slower due to the absence of an activating functional group. Namely, attempts at condensation of dialdehyde **5** with dimethyl malonate, dimedone, 1,3-indandione, 2,2-dimethyl-1,3-dioxane-4,6-dione or 1,2,3,3-tetramethyl-3*H*-indolium perchlorate, triphenyl phosphonium ylides (Wittig reagents), and *N*-methyl-4-picolinium bromide failed to give the expected products.

We next considered the transformation of dialdehyde **5** into 2,5-bis(ethynyl)pyrrolo[3,2-*b*]pyrrole. Such transformations have been achieved with good yields for electron-rich aromatic aldehydes using the Bestmann–Ohira reagent (BOR), which is a 10% solution of dimethyl(1-diazo-2-oxopropyl)phosphonate in acetonitrile.^22^ When used for transformation of **5**, the progress of the reaction was again hampered by the low reactivity of the aldehyde, and 6 equiv of BOR was needed in order to obtain satisfactory conversion to the desired 2,5-bis(ethynyl)pyrrolo[3,2-*b*]pyrrole (Scheme 2).^23^ Because of the light sensitivity of this material, it was used directly in the Sonogashira reaction to further expand the π-system leading to dyes **7** and **8**.

**Scheme 2:**
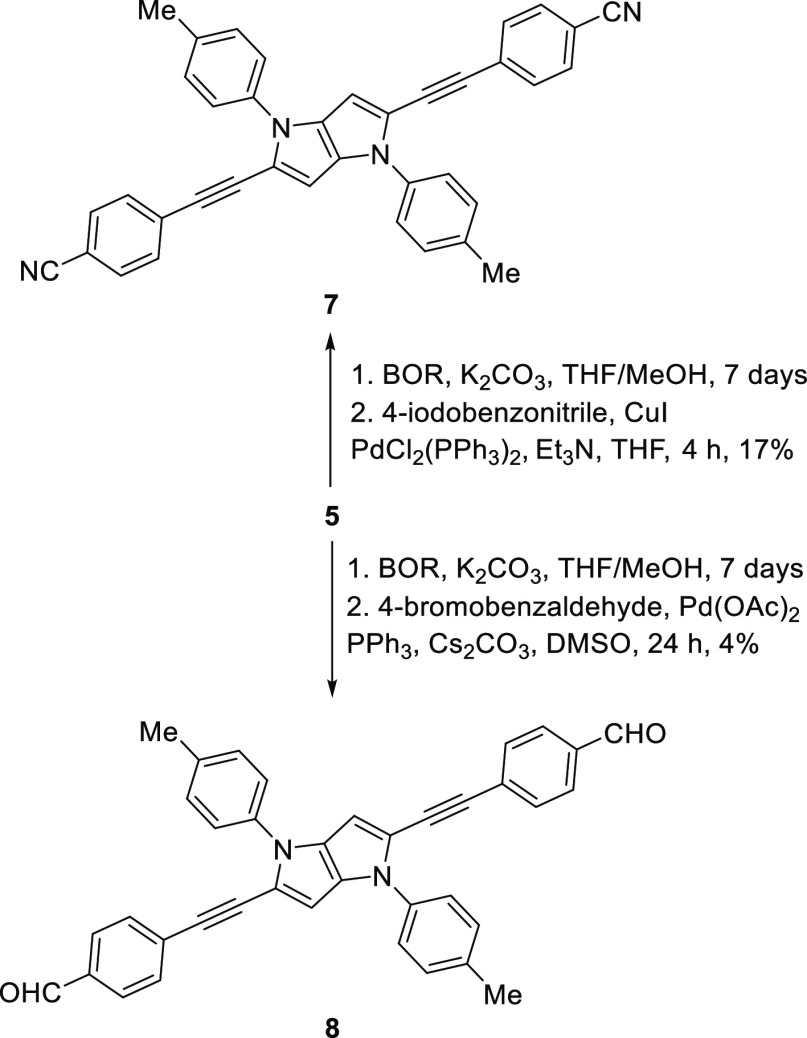
Synthesis of Pyrrolo[3,2-*b*]pyrroles **7** and **8**

The photophysical properties of pyrrolo[3,2-*b*]pyrroles **6**–**8** were measured in cyclohexane, toluene, dichloromethane, tetrahydrofuran, and acetonitrile (Figure 1, Table 1, and Table S5). As expected, newly synthesized dyes **7** and **8** have their absorption and emission bathochromically shifted compared to traditional tetraaryl derivatives as a result of expansion of their π-electron system. A noticeable, >100 nm red-shift is shown by compound **6** bearing strongly electron-withdrawing cyano substituents when compared to parent TAPP **4**. The fluorescence quantum yields (φ_fl_) of dyes **7** and **8** exceed 40%, which correspond very well to those reported earlier for this class of dyes. In the case of dye **6**, however, φ_fl_ is ca. 1%, which can be attributed to rotational relaxation of the excited state. All prepared dyes exhibit moderate solvatochromism, with 13 and 17 nm hypsochromic shifts registered for **7** and **8**, respectively, when going from cyclohexane to acetonitrile (Figures S11–S13).

**Table 1:** Spectroscopic Properties of Dyes **6**–**8** in Different Solvents

compd	solvent	λ_abs_^max^ (nm)	ε_max_ (M^–1^ cm^–1^)	λ_em_^max^ (nm)	Stokes shift (cm^–1^)	φ_fl_	2λ_abs_^max^ (nm)	λ_TPA_^max^ (nm)	σ_TPA_^max^ (GM)	σ_TPA_^max^ φ_fl_ (GM)
**6**	CHX	501	92200	509	314	0.009^a^				
		472	59700							
	DCM	516	73000	540	861	0.013^a^	1032	648	505 ± 95 (1340 ± NA)	6.6
		481	56000				962	577		17
	ACN	505	59100	540	1280	0.007^a^				
		481	52100							
**7**	CHX	443	116800	451	400	0.51^b^				
		419	84800							
	DCM	441	81700	486	2100	0.06^b^	882	721	1050 ± 190	63
	ACN	430	80100	504	3420	0.01^b^				
**8**	CHX	459	92200	469	564	0.53^b^				
		435	68600							
	DCM	455	71000	566	4310	0.02^b^	910	770	825 ± 160	17
	ACN	442	66200	581	5410	0.002^b^				

a Determined with fluorescein in 0.1 M NaOH as a standard.

b Determined with coumarin 153 in ethanol as a standard. CHX – cyclohexane, DCM – dichloromethane, ACN – acetonitrile.

**Figure 1:**
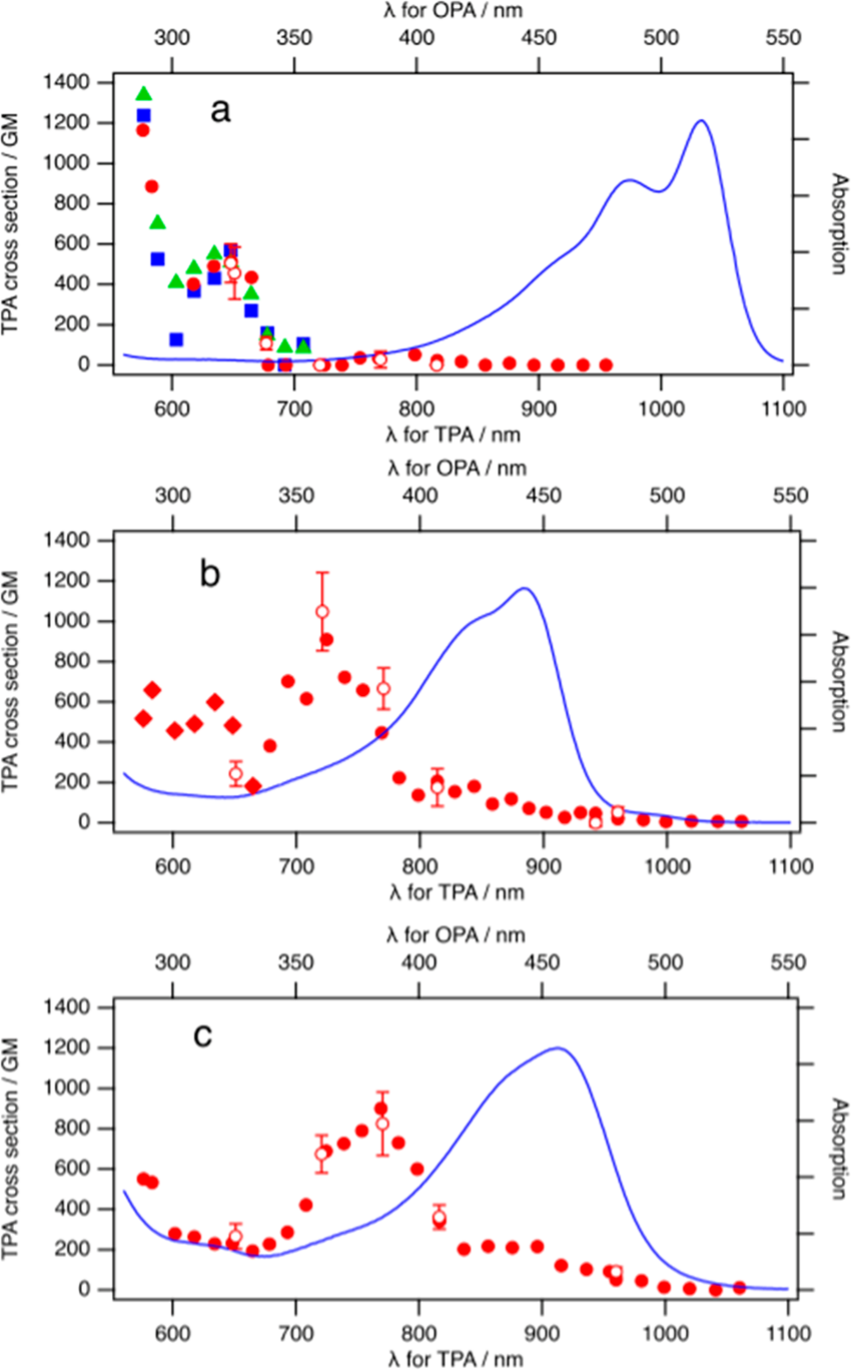
TPA spectrum of **6** (a), **7** (b), and **8** (c) in dichloromethane measured at a fixed incident power (filled symbols): 0.25 (square), 0.40 (circle and diamond), and 0.50 mW (triangle) measured by varying the power (open circle with error bar) together with the OPA spectrum (solid line). The bottom and left axes relate to the TPA and the top and right axes to OPA. The bottom and top axes are scaled so that the transition energies of TPA and OPA are located in the same position. The data to which saturable absorption analysis was applied is shown by diamonds.

Inspection of the results suggests a polarized character of the ground state. More spectacular changes are observed on the emission spectra of **7** and **8**, with strong positive solvatofluorochromism expressed by an over 100 nm bathochromic shift observed for compound **8**. Altogether, this results in an increase of Stokes shift by a factor of ca. 10 when going from nonpolar cyclohexane to polar acetonitrile.

TPA properties of **6**–**8** were studied by experimental and quantum-chemical calculations (see the Supporting Information for details). The measurements showed that pyrrolo[3,2-*b*]pyrrole dyes **7** and **8** have a reasonably large TPA cross-section at wavelengths around 750 nm (Figure 1). The peak values observed are 1050 ± 190 GM for **7** at 720 nm and 825 ± 160 GM for **8** at 770 nm. These TPA peaks have transition energies (i.e., twice the photon energies) located at lower energies than those of one-photon absorption. A weaker but non-negligible TPA was observed at twice the wavelength of the OPA peak (800–900 nm for **7** and 850–950 nm for **8**). These weak TPA bands can be explained by partial relaxation of forbidden a TPA transition to the OPA-allowed excited states due to vibronic coupling.

Compared to these two, the TPA peak for **6** was found to be significantly blue-shifted and with a relatively smaller magnitude of the peak cross-section (505 ± 95 GM at 648 nm). At short wavelengths below 600 nm, the spectral magnitude showed a drastic increase as incident wavelength was decreased and reached ca. 1300 GM at 577 nm.

The observed blue-shift of the TPA peak and red-shift of the OPA peak of **6** results in exceptionally wide separation of the transition energy between the OPA and TPA transitions as shown in Figure 1.

Spectrum simulation by quantum-chemical calculations at the CAM-B3LYP/6-31+G(d) level of theory (see details in the Supporting Information) successfully reproduced the main features of the OPA and TPA spectra for **6**–**8** (Figures S5–S7) with the usual level of overestimation of the transition energy by 0.24–0.28 eV. The decomposed TPA spectra by destination state showed that the main TPA bands for **7** and **8** were assigned to the transition to the S_2_ excited state, while that for **6** was assigned to the transition to S_4_ (Figure S8). The drastic increase at wavelengths shorter than the peak of **6** can be explained by the strong TPA transition to S_9_ together with a weaker one to S_6_. For all dyes, the lowest energy OPA band is assigned to the transition to S_1_ (HOMO → LUMO) as listed in Table 2. The electronic configuration of all the TPA excited states (S_2_ states of **7** and **8** and S_4_ states of **6**) was found to be HOMO → LUMO+1.

**Table 2:** Calculated Results of the Excited States (*S*_*n*_) Involved in the Major Transitions of OPA and TPA for Dyes **6**–**8**^a^

dye	*S*_*n*_	*E* (eV)	λ (nm)	*f*	config (amplitude)
**6**	1	2.88	431	1.90	H → L (0.689)
	2	3.34	371	0.30	H-1 → L (0.691)
	**4**	**4.26**	**291**	**0.00**	**H → L + 1 (0.648)**
	**9**	**4.83**	**257**	**0.00**	**H-6 → L (0.668)**
**7**	1	3.17	391	3.03	H → L (0.670)
	**2**	**4.00**	**310**	**0.00**	**H → L + 1 (0.643)**
	3	4.13	300	0.25	H-1 → L (0.664)
	**8**	**4.96**	**250**	**0.00**	**H-2 → L (0.437)**
**8**	1	3.08	403	2.99	H → L (0.663)
	**2**	**3.81**	**326**	**0.00**	**H → L + 1 (0.647)**
	5	4.08	304	0.22	H-1 → L (0.657)
	**9**	**4.08**	**257**	**0.00**	**H-2 → L (0.507)**

a Transition energy *E*, transition wavelength λ, oscillator strength *f*, and electronic configuration (and its amplitude). H stands for HOMO and L stands for LUMO. Bold type represents the TPA excited state.

Two-photon transition to S_2_ for **7** and **8** is solely mediated by S_1_ as an intermediate state (S_0_ → (S_1_) → S_2_). The transition dipole moments from S_0_ to S_1_ (μ_10_) and S_1_ to S_2_ (μ_21_) are μ_10_ = 16.3 D and μ_21_ = 17.2 D with the crossing angle (θ) of 1.7° for **7** and μ_10_ = 16.5 D and μ_21_ = 19.7 D with θ = 1.4° for **8**. These large transition dipole moments with small angles result in efficient TPA.^24^ These properties originate from the large overlap of the molecular orbitals (HOMO → LUMO for μ_10_ and LUMO → LUMO+1 for μ_21_), distributing in the same direction along the long axis of the molecule for both **7** and **8** (Figure 2). The same was observed for **6**. The dominant intermediate was also found to be the S_1_ state for its two-photon transition (i.e., S_0_ → (S_1_) → S_4_).

**Figure 2:**
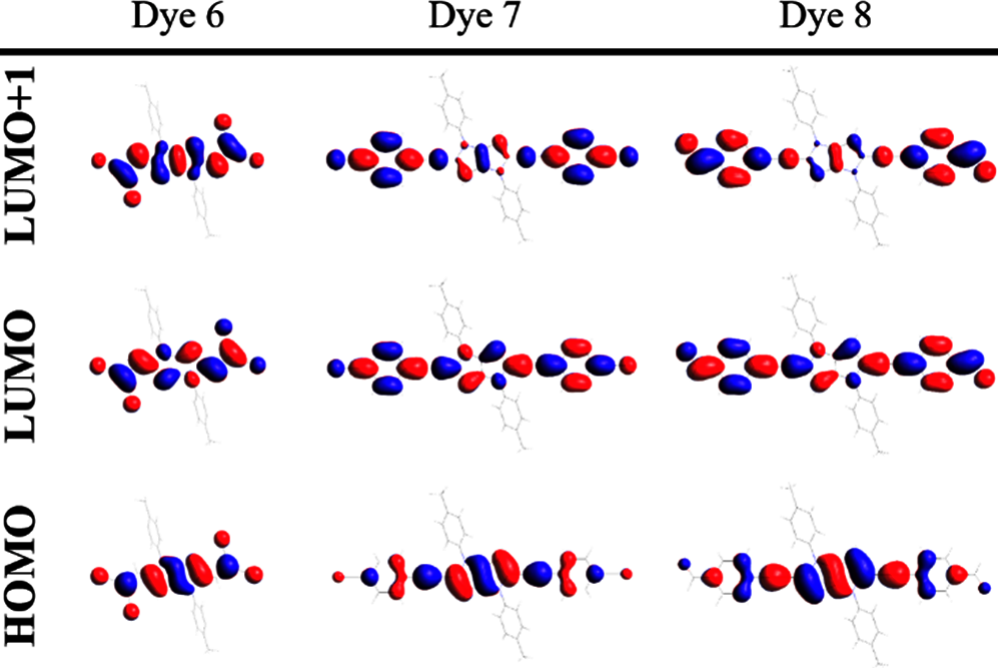
Isosurface plots of selected molecular orbitals involved in the lowest one- and two-photon absorption excitations of **6**–**8**.

The *N*-phenyl rings are twisted by 69° for **6** and 54° for **7** and **8** with respect to the pyrrolo[3,2-*b*]pyrrole plane (Figure S9). This gives virtually zero overlap between the orbitals and makes them a dark state for both OPA and TPA. In addition, the experimentally observed wide separation of the transition energy between the OPA (516 nm, 2.40 eV) and TPA (650 nm/2, 3.81 eV) peaks of **6** (by 1.41 eV) can be explained by the order of the excited states (where TPA occurs to S_4_) and the shorter π-conjugation length.

In conclusion, thiazole is shown to be a CHO equivalent in the first ever synthesis of 2,5-diformylpyrrolo[3,2-*b*]pyrrole, which in turn is an excellent building block for the preparation of 2,5-diethynylpyrrolo[3,2-*b*]pyrrole. Centrosymmetric, quadrupolar dyes prepared from these two building blocks have relatively large TPA cross sections such as 800–1000 GM at ca. 750 nm for phenylethynyl π-extended derivatives. As for the dicyanoethenyl containing dye, the TPA bands were blue-shifted (680 nm) and showed a drastic increase to ca. 1300 GM. Quantum-chemical calculations revealed that the separation between excited states responsible for one-photon and two-photon absorption is exceptionally large in this case, which can be traced to bridging the strongly electron-withdrawing CH=C(CN)_2_ groups with the exceptionally electron-rich pyrrolo[3,2-*b*]pyrrole core.

## Supporting Information Available

Data associated with this article, including experimental procedures, compounds characterization, steady-state absorption and emission along with the two-photon absorption details, electrochemical details and computational analysis details (PDF)
FAIR data, including the primary NMR FID files, for compounds **5**-**8** (ZIP)
