# Supplementary material for: From 2,5-Diformyl-1,4-dihydropyrrolo[3,2-b]pyrroles to Quadrupolar, Centrosymmetric Two-Photon-Absorbing A–D–A Dyes
Source: Org Lett. 2022 Mar 28;24(13):2551–5. doi: 10.1021/acs.orglett.2c00718 (PMC9003575; doi:10.1021/acs.orglett.2c00718)
Supplement: Supplementary file 1 — ol2c00718_si_001.pdf [file ol2c00718_si_001.pdf]

Supporting information for

From 2,5-Diformyl-1,4-dihydropyrrolo[3,2-*b*]pyrroles to Quadrupolar, Centrosymmetric Two-photon-absorbing A-D-A Dyes

Paweł Kowalczyk,[a] Mariusz Tasior,[a] Shuhei Ozaki,[b,c] Kenji Kamada,[b,c]* Daniel T. Gryko[a]*

[a] *Institute of Organic Chemistry, Polish Academy of Sciences, Kasprzaka 44/52, 01-224 Warsaw, Poland.*
[b] *NMRI, National Institute of Advanced Industrial Science and Technology (AIST), Ikeda, Osaka 563-8577, Japan*
[c] *Department of Chemistry, Graduate School of Science and Technology, Kwansei Gakuin University, Sanda 669-1337, Japan*

Table of contents

1. General information 2
2. Synthesis 4
3. Two-photon absorption studies 7
4. Quantum chemical calculations 14
5. Spectroscopic properties 20
6. Absorption and emission spectra 21
8. ^{1}H and ^{13}C NMR spectra 24
9. References 29

1. General information

All chemicals were used as received, unless otherwise noted. All reported NMR spectra were recorded on a 500 MHz spectrometer unless otherwise noted. Chemical shifts (δ; ppm) for ^{1}H and ^{13}C NMR were determined with TMS as the internal reference. *J* values are given in Hz. Absorption and fluorescence spectra were recorded in cyclohexane, toluene, tetrahydrofuran, dichloromethane and acetonitrile. Mass spectra were obtained with an EI ion source and EBE double focusing geometry mass analyzer or from a spectrometer equipped with an electro-spray ion source with Q-TOF type mass analyzer.

Two-photon absorption spectral measurements.

Two-photon absorption (TPA) spectra were measured by the open-aperture Z-scan method[1] with a femtosecond optical parametric amplifier (Spectra-Physics TOPAS Prime) as the wavelength-tunable light source. The setup for the measurements and the analysis are only described briefly here, because they have been previously reported in detail.[2] In the Z-scan measurements, transmittance is recorded as function of sample position, scanned along the beam propagation direction of the focused laser beam. Typical examples of the open-aperture Z-scan traces are shown in Figs. S1–3 as examples. The transmittance is normalized to that at a sample position far from the focal point and is theoretically described as,

$$T(\zeta) = T_N \frac{1}{\sqrt{\pi}\, q(\zeta)} \int_{-\infty}^{+\infty} \ln\left[1 + q(\zeta)\, e^{-x^2}\right] dx \tag{S1}$$

for a two-photon absorber excited by spatially and temporally Gaussian pulses. Here, $T_N = \exp(-\alpha L)$ is the linear transmittance of the sample, α is the linear absorption coefficient, L is the physical path length of the sample, and ζ is the normalized sample position $\zeta = z/z_R$ with the Rayleigh length z_R. $q(\zeta)$ is the on-axis two-photon absorbance $q(\zeta) = q_0/(1+\zeta^2)$ with the on-axis two-photon absorbance at the sample position $q(0)$=q_0. This q_0 is related to the two-photon absorption coefficient β by,

$$q_0 = \beta I_0 L \tag{S2}$$

where I_0 is the on-axis peak intensity of the incident pulse. For the measurements performed using a fixed incident power (i.e., a fixed I_0), β was calculated by assuming the proportionality relation of eq. S2. Practically, artifacts or unwanted processes may cause apparent deviation from proportionality, so we also recorded the incident-power dependence (i.e., by changing I_0)

at some wavelengths to check this relationship. In this case, β was determined from the slope of the plot of q_0 against I_0. The data points obtained by this method were shown with error bars in Fig. 2 in the main text. Finally, the TPA cross section $\sigma^{(2)}$ was obtained from β by using the convention $\sigma^{(2)} = h\nu\beta/N$, where $h\nu$ is the photon energy of the excitation pulse and N is the number density of the sample molecule calculated from the concentration.

The dyes were dissolved in spectroscopic grade dichloromethane (Fuji Film-Wako). Solutions were then held in 2-mm cuvettes whose path length is short enough against z_R (7–10 mm) to satisfy the thin sample condition for eq. S1. To avoid unwanted effects such as interference by photoproducts etc., the samples were stirred by a micro rotor put in the cell during the measurements. No significant degradation was observed for the samples after Z-scan measurements, which is confirmed by UV-vis measurements. For calibration of day-by-day fluctuation of the measured values, inhouse standard materials were measured at the same time with the samples (MPPBT in dimethyl sulfoxide[3,4]).

The open aperture z-scan traces at 650 nm or shorter for **7** showed saturable absorption (SA) as shown in Fig. S4. These z-scan traces were analyzed by phenomenological modeling of SA as

$$\alpha = \alpha\big(I(\zeta)\big) = \frac{\alpha_0}{(1+I(\zeta)/I_S)} \tag{S3}$$

where I_S is saturation intensity and $I(\zeta) = I_0/(1+\zeta^2)$.[5]

Quantum chemical calculations.

Molecular structures were optimized at the CAM-B3LYP/6-31+G(d) level of theory. The appropriateness of the obtained structures were checked by the absence of negative frequency in frequency calculations. The optimized structures are shown in Fig. S9. Transition dipole moments and transition energies, and permanent dipole moments were calculated for the lowest 20 excited states using the Tamm-Dancoff Approximation (TDA)[6] with the same level of theory (CAM-B3LYP/6-31+G(d)). All calculations were performed in the Gaussian program suit.[7] Solvent effects were considered by the polarizable continuum model (PCM) for dichloromethane for all calculations In this model, the bulk effect induced by a reaction field is considered. The solvent is modeled as a continuous medium with a dielectric constant, and the solute molecule is placed inside a cavity in the media. The reaction field is formed by polarization of the solvent medium surrounding the cavity, so it is also applied to the solute molecule in the cavity, in addition to the external field of incident light. The shape of the cavity is overlapping

spheres in PCM in contrast to the single sphere in the Onsager model.[8] Calculated features of the lowest 10 excited states of **6**–**8** are listed in Tables S1–S3, respectively.

For postprocessing of the TPA spectrum simulation, we used a homemade program on Igor Pro (Wavemetrics Inc.) based on reported procedures.[9] The spectral simulation by this method gives relatively good agreement of the calculated TPA cross section for the molecules with short π-conjugation (containing a few double bonds) but overestimates for longer π-conjugation by one order of magnitude of more. A rigorous explanation of the overestimation is not easy, but it often originates from the overestimation of transition dipole moments. The overestimation was enhanced for the higher-order (multiphoton) absorption process because the fourth power of transition dipole moments is necessary for TPA while only the square is needed for linear optical properties (OPA). The relaxation constants of OPA and TPA were set to 0.07 eV so that the OPA peak in the simulated spectrum matched to the 0-0 peak of the experimental one. The choice of the relaxation constants also directly affects the magnitude of the calculated TPA cross section because the height of the peak is inversely proportional to the square of the relaxation constant. Therefore, the comparison in absolute value to experiential results is very difficult. We would like to emphasize that the simulation results are useful for comparison of the relative shape of the spectra and clarifying the nature of the transition (allowed/forbidden).

2. Synthesis

Synthesis of 1,4-bis(4-methylphenyl)-2,5-bis(thiazol-2-yl)-1,4-dihydropyrrolo[3,2-*b*]pyrrole (4)

Glacial acetic acid (7.5 mL), toluene (7.5 mL), 2-thiazolecarboxaldehyde (**1**, 880 μL, 10 mmol) and *p*-toluidine (**2**, 1.07 g, 10 mmol) were placed in a 50 mL round-bottom flask equipped with a magnetic stir bar. The mixture was stirred at 90 °C in an oil bath for 30 min. After that time, $Fe(ClO_4)_3 \cdot xH_2O$ (166 mg, ca. 0.5 mmol) was added, followed by diacetyl (**3**, 440 μL, 5 mmol). The resulting mixture was stirred at 90 °C for another 3 h and then cooled to room temperature. The solvents were evaporated off and the residue dissolved in hot ACN. After naturally cooling to room temperature it was left overnight in a refrigerator. The resulting precipitate was filtered off, washed with cold ACN and dried under high vacuum.

Yellow solid. Yield: 780 mg (34%). Spectroscopic properties were in agreement with the literature data.[10]

Synthesis of 2,5-bis(formyl)-1,4-bis(4-methylphenyl)-1,4-dihydropyrrolo[3,2-*b*]pyrrole (5)

Alkylation: Compound **4** (1560 mg, 3.4 mmol) was placed in round-bottom pressure flask equipped with magnetic stir bar. The solid was suspended in 57 mL of dry DMF under an inert atmosphere, followed by addition of methyl triflate (1.6 mL, 7 mmol). The resulting mixture was stirred at 130 °C in an oil bath for 24 h. The solvent was evaporated off and the resulting oil was dissolved in hot DCM. After naturally cooling to room temperature it was left overnight in a refrigerator. The resulting precipitate was filtered off and washed with cold DCM. After drying under high vacuum, this material was used in the next step without any further purification.

Reduction and cleavage: Alkylated TAPP (1085 mg, 1.4 mmol) was dissolved in 30 mL of MeOH and 50 mL of THF. $NaBH_4$ (535 mg, 14 mmol) was added in a few portions and the reaction mixture was stirred for 30 min at room temperature. Subsequently, 2 mL of acetone was added and the solvents evaporated under reduced pressure. The residue was extracted with DCM and brine. The combined organic layers were dried over Na_2SO_4 and evaporated. The residue was dissolved in an ACN/THF mixture (150 mL/50 mL) and 30 mL of 0.05 M (pH =7) phosphate buffer was poured into this mixture. Then, $AgNO_3$ (480 mg, 2.8 mmol) in 20 mL of water was added. After 30 min, another identical solution of $AgNO_3$ was added. After 24h, 1.2 mL of Et_3N was added, and after another 30 min brine was added. The reaction mixture was filtered through a pad of Celite and the filtrate was extracted with DCM. The combined organic layers were dried over Na_2SO_4 and evaporated. The residue was purified by column chromatography (SiO_2, DCM).

Yellow solid. 200 mg (41 %). mp 208-209 °C. ^{1}H NMR (500 MHz, $CDCl_3$): δ 9.69 (s, 2H), 7.36 - 7.31 (m, 8H), 6.89 (s, 2H), 2.45 (s, 6H) ppm; ^{13}C NMR (126 MHz, $CDCl_3$): δ 180.5, 138.5, 137.9, 135.3, 134.9, 130.0, 125.6, 101.4, 21.2 ppm. HRMS (EI) m/z: $[M]^{\cdot +}$ Calcd for $C_{22}H_{18}N_2O_2$ 342.1368; Found 342.1375.

Synthesis of compound 6

A dried Schlenk flask was charged with **5** (41 mg, 120 μmol) and an ACN/THF (3 mL/1.5 mL) mixture, followed by malononitrile (23mg, 350 μmol). The reaction mixture was stirred at rt for 4h. The resulting precipitate was filtered off and recrystallized from $CHCl_3$/MeOH.

Red solid. 53 mg (100 %). mp 270 °C (decomp.). ^{1}H NMR (500 MHz, $CDCl_3$): δ 7.41 (d, J = 8.0 Hz, 4H), 7.39 (s, 2H), 7.35 (s, 2H), 7.22 (d, J = 8.0 Hz, 4H), 2.50 ppm (s, 6H); ^{13}C NMR (126 MHz, $CDCl_3$)

δ 144.5, 140.1, 137.8, 136.3, 132.3, 131.1, 126.8, 114.5, 113.7, 97.8, 76.1, 21.2 ppm. HRMS (EI) m/z: $[M]^{\cdot+}$ Calcd for $C_{28}H_{18}N_6$ 438.1593; Found 438.1592.

Synthesis of compound 7

A dried Schlenk flask was charged with **5** (200 mg, 585 μmol) and K_2CO_3 (323 mg, 2.3 mmol). Dry THF (15 mL) and dry MeOH (15 mL) were then added. After three vacuum/argon cycles, dimethyl(1-diazo-2-oxopropyl)phosphonate (247 mg, 1.3 mmol) was added and the reaction mixture was stirred at room temperature for 2 days. More dimethyl(1-diazo-2-oxopropyl)phosphonate (247 mg, 1.3 mmol) was added, and reaction mixture was stirred at room temperature for another 2 days. Further addition of dimethyl(1-diazo-2-oxopropyl)phosphonate (247 mg, 1.3 mmol) and stirring at room temperature for another 3 days led to complete conversion of starting material (attempts to accelerate the reaction by increasing the temperature resulted in the decomposition of the product). The solvents were evaporated off under reduced pressure. The residue was dissolved in DCM and extracted with saturated $NaHCO_{3(aq)}$. The combined organic layers were extracted with brine, then dried over $MgSO_4$ and evaporated. The resulting residue was dried under high vacuum and used in next step without any further purification.

A dried Schlenk flask was charged with CuI (6 mg, 33 μmol), $PdCl_2(PPh_3)_2$ (23mg, 33 μmol) and 4-iodobenzonitrile (282 mg, 1.23 mmol). The reagents were dissolved in a mixture of dry THF (3.5 mL) and dry Et_3N (1.7 mL). A solution of crude 2,5-bis-(ethynyl)pyrrolopyrrole in 5 mL of dry THF was then added dropwise over the period of 1h at room temperature and under argon. The reaction mixture was heated in an oil bath at reflux for 4h. The mixture was cooled to room temperature and solvent evaporated off under reduced pressure. The residue was purified by column chromatography (SiO_2, DCM/hexanes, 1:1) and recrystallized from $CHCl_3$/MeOH.

Red solid. 53 mg (17 %). mp 290 °C (decomp.). ^{1}H NMR (500 MHz, $CDCl_3$): δ 7.57 (d, *J* = 8.0 Hz, 4H), 7.56 (d, *J* = 8.0 Hz, 4H), 7.39 (d, *J* = 8.0 Hz, 4H), 7.34 (d, *J* = 8.0 Hz, 4H), 6.63 (s, 2H), 2.47 ppm (s, 6H); ^{13}C NMR (126 MHz, $CDCl_3$) δ 136.5, 136.1, 132.0, 130.8, 130.3, 129.7, 128.1, 123.9, 119.1, 118.6, 110.1, 100.4, 94.4, 88.1, 21.1 ppm. HRMS (EI) m/z: $[M]^{\cdot+}$ Calcd for $C_{38}H_{24}N_4$ 536.2001; Found 536.2004.

Synthesis of compound 8

Crude 2,5-bis-ethynylpyrrolopyrrole was prepared as described for **7**.

A dried Schlenk flask was charged with 4-bromobenzaldehyde (238 mg, 1.3 mmol), Cs_2CO_3 (400 mg, 1.2 mmol), $Pd(OAc)_2$ (33 mg, 146 μmol) and PPh_3 (153 mg, 585 μmol). A solution of crude 2,5-bis-(ethynyl)pyrrolopyrrole in 8 mL of dry DMSO was then added. The mixture was degassed and refilled with argon 3 times. The reaction mixture was stirred at 80 °C in an oil bath for 24h after which it was cooled to room temperature and the solvent evaporated under reduced pressure. The residue was purified by column chromatography (SiO_2, DCM/hexanes) and recrystallized from $CHCl_3$/MeOH.

Red solid. 13 mg (4 %). mp 257 °C (decomp.). ^{1}H NMR (500 MHz, $CDCl_3$) δ 9.97 (s, 2H), 7.81 (d, *J* = 8.2 Hz, 4H), 7.59 (d, *J* = 8.2 Hz, 4H), 7.47 (d, *J* = 8.2 Hz, 4H), 7.34 (d, *J* = 8.2 Hz, 4H), 6.64 (s, 2H), 2.47 ppm (s, 6H); ^{13}C NMR (126 MHz, $CDCl_3$) δ 191.2, 136.4, 136.2, 134.9, 130.8, 130.2, 129.7, 129.6, 129.5, 123.9, 119.2, 100.4, 95.2, 87.9, 21.1 ppm. HRMS (EI) m/z: $[M]^{\cdot+}$ Calcd for $C_{38}H_{26}N_2O_2$ 542.1994; Found 542.2011.

3. Two-photon absorption studies

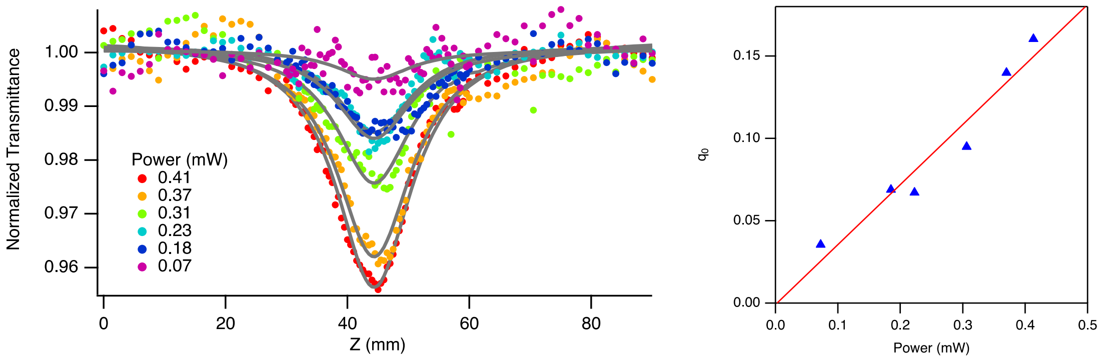

Figure S1. (left) Open-aperture Z-scan traces of **6** in dichloromethane (0.43 mM) measured at 651 nm at different incident powers (symbols) with theoretical fits (grey curves). (right) The corresponding plot of the two-photon absorbance q_0 obtained from the curve fits with eq. S1 against the incident power in the left panel.

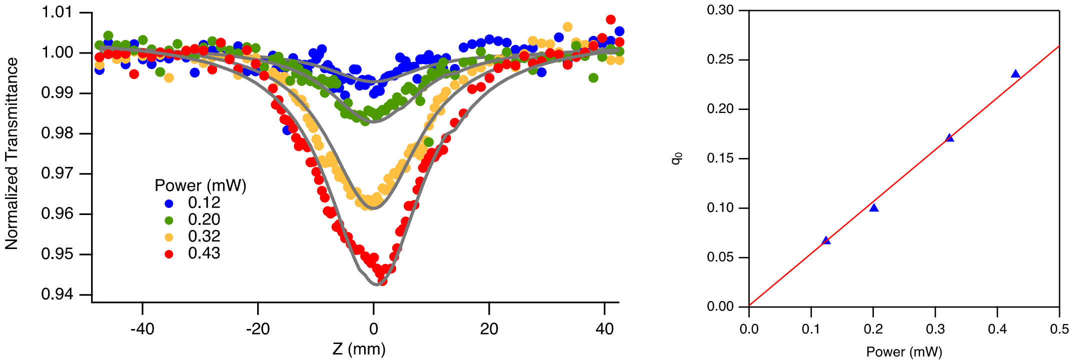

Figure S2. (left) Open-aperture Z-scan traces of **7** in dichloromethane (0.47 mM) measured at 721 nm at different incident powers (symbols) with theoretical fits (grey curves). (right) The corresponding plot of the two-photon absorbance q_0 obtained from the curve fits with eq. S1 against the incident power in the left panel.

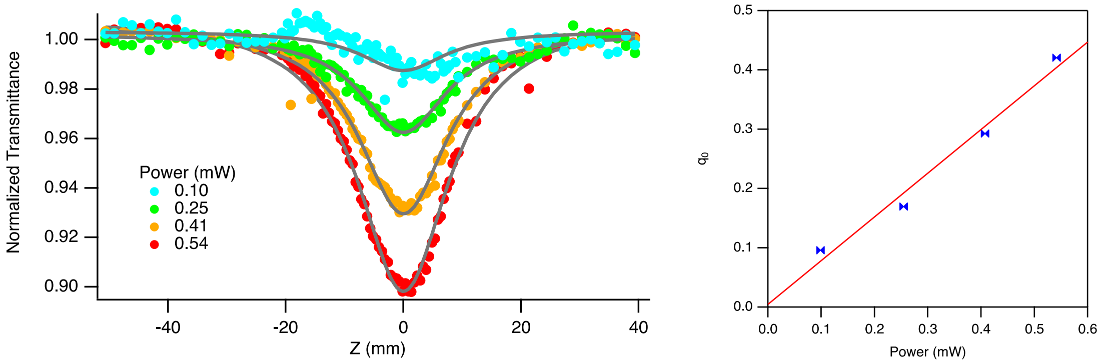

Figure S3. (left) Open-aperture Z-scan traces of **8** in dichloromethane (0.70 mM) measured at 770 nm at different incident powers (symbols) with theoretical fits (grey curves). (right) The corresponding plot of the two-photon absorbance q_0 obtained from the curve fits with eq. S1 against the incident power in the left panel.

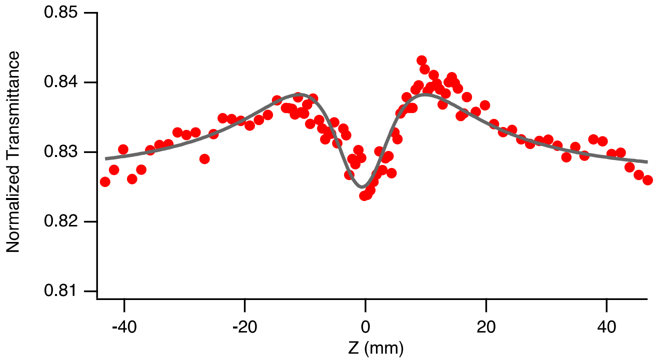

Figure S4. Open-aperture Z-scan trace of **7** (dots) in dichloromethane (0.52 mM) measured at 583 nm (incident power of 0.4 mW) with theoretical fits considering saturable absorption (grey curves) with eq. S3.

(a)

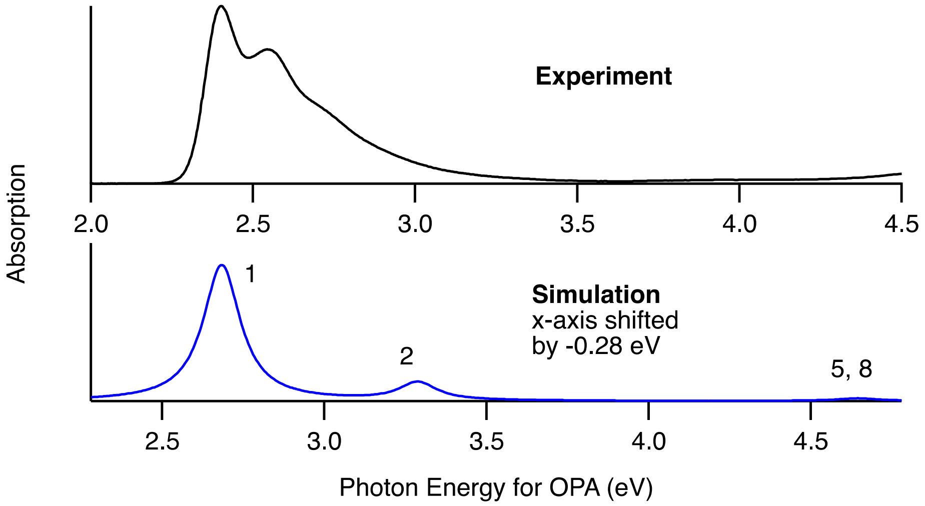

(b)

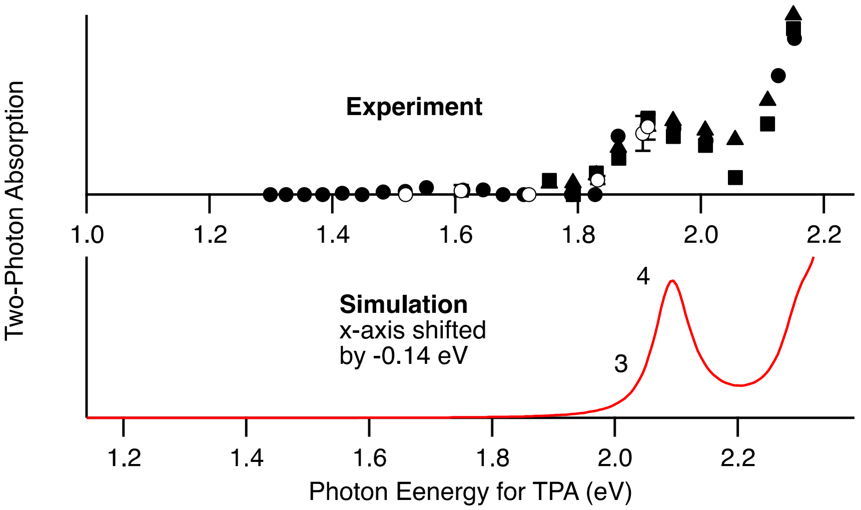

Figure S5. Comparison of the experimental and simulated spectra for OPA (a) and TPA (b) of Dye **6** in dichloromethane. The location (not the value) of x-axes of the simulation spectra is shifted (by -0.28 eV for OPA and by -0.14 eV for TPA) so that the simulated OPA peak comes to the same horizontal position on the sheet as the experimental one and one can compare them easier by compensating the overestimation of the transition energy of the excited state by the calculations. The numbers by the spectral peaks designated the destination excited state of the transition. The calculation level employed was TDA-CAM-B3LYP/6-31+G(d) with PCM. For the details of the calculation methods, see Experimental in SI. The symbols in the experimental TPA spectrum (from Fig. 2) means the data points measured at a fixed incident power (filled square: 0.25 mW, filled circle: 0.4 mW, filled triangle: 0.50 mW) and by varying it (open circle with error bar: 0.1–0.4 mW), respectively.

(a)

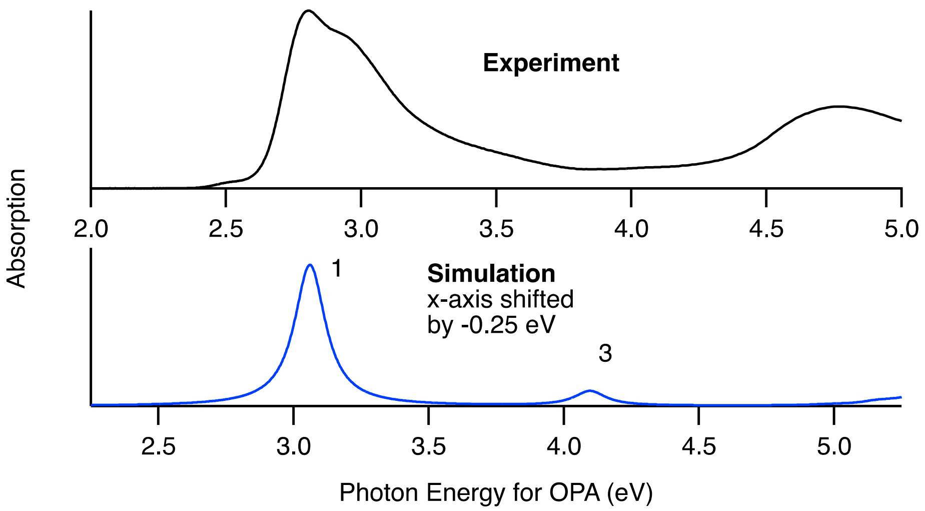

(b)

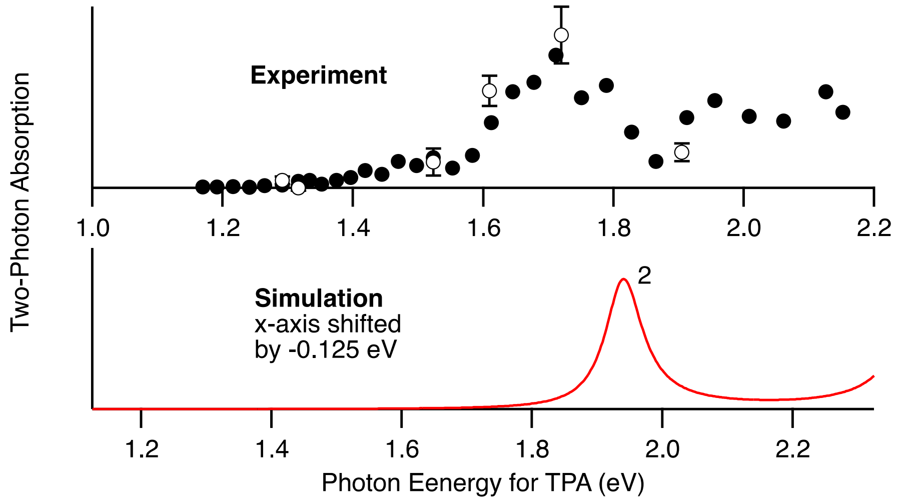

Figure S6. Comparison of the experimental and simulated spectra for OPA (a) and TPA (b) of Dye **7** in dichloromethane. The location (not the value) of x-axes of the simulation spectra is shifted (by -0.25 eV for OPA and by -0.125 eV for TPA) so that the simulated OPA band comes to the same horizontal position on the sheet as the experimental one and one can compare them easier by compensating the overestimation of the transition energy of the excited state by the calculations. The numbers by the spectral peaks designated the destination excited state of the transition. The calculation level employed was TDA-CAM-B3LYP/6-31+G(d) with PCM. For the details of the calculation methods, see Experimental in SI. The filled and open circles in the experimental TPA spectrum (from Fig. 2) means the data points measured at a fixed incident power (0.4 mW) and by varying it (0.1–0.4 mW), respectively.

(a)

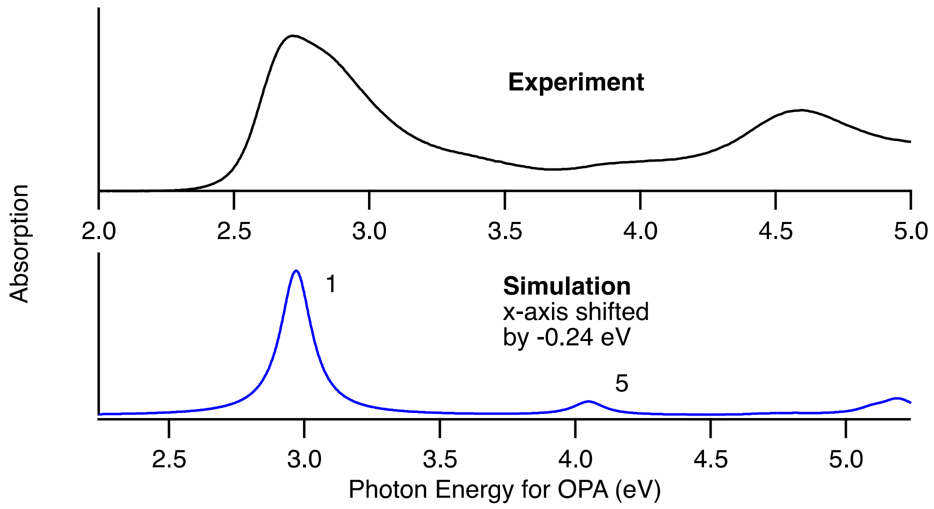

(b)

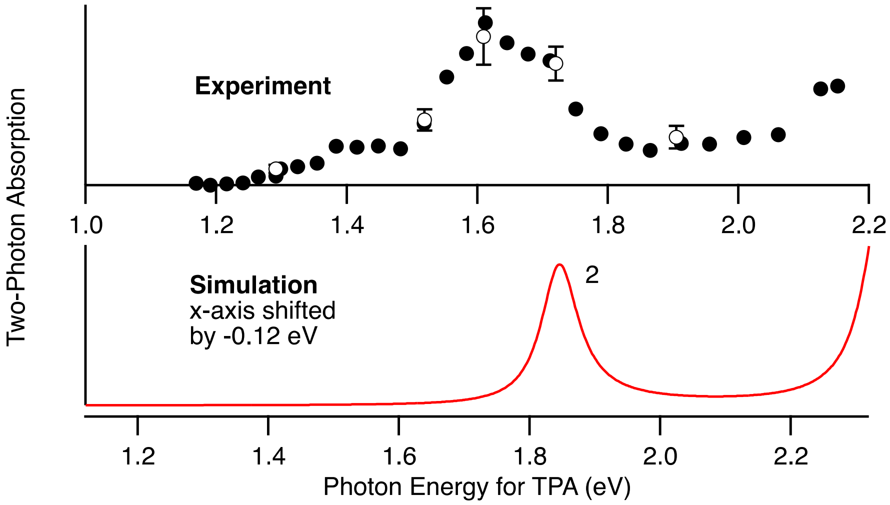

Figure S7. Comparison of the experimental and simulated spectra for OPA (a) and TPA (b) of Dye **8** in dichloromethane. The location (not the value) of x-axes of the simulation spectra is shifted (by -0.24 eV for OPA and by -0.12 eV for TPA) so that the simulated OPA band comes to the same horizontal position on the sheet as the experimental one and one can compare them easier by compensating the overestimation of the transition energy of the excited state by the calculations. The numbers by the spectral peaks designated the destination excited state of the transition. The calculation level employed was TDA-CAM-B3LYP/6-31+G(d) with PCM. For the details of the calculation methods, see Experimental in SI. The filled and open circles in the experimental TPA spectrum (from Fig. 2) means the data points measured at a fixed incident power (0.4 mW) and by varying it (0.1–0.4 mW), respectively.

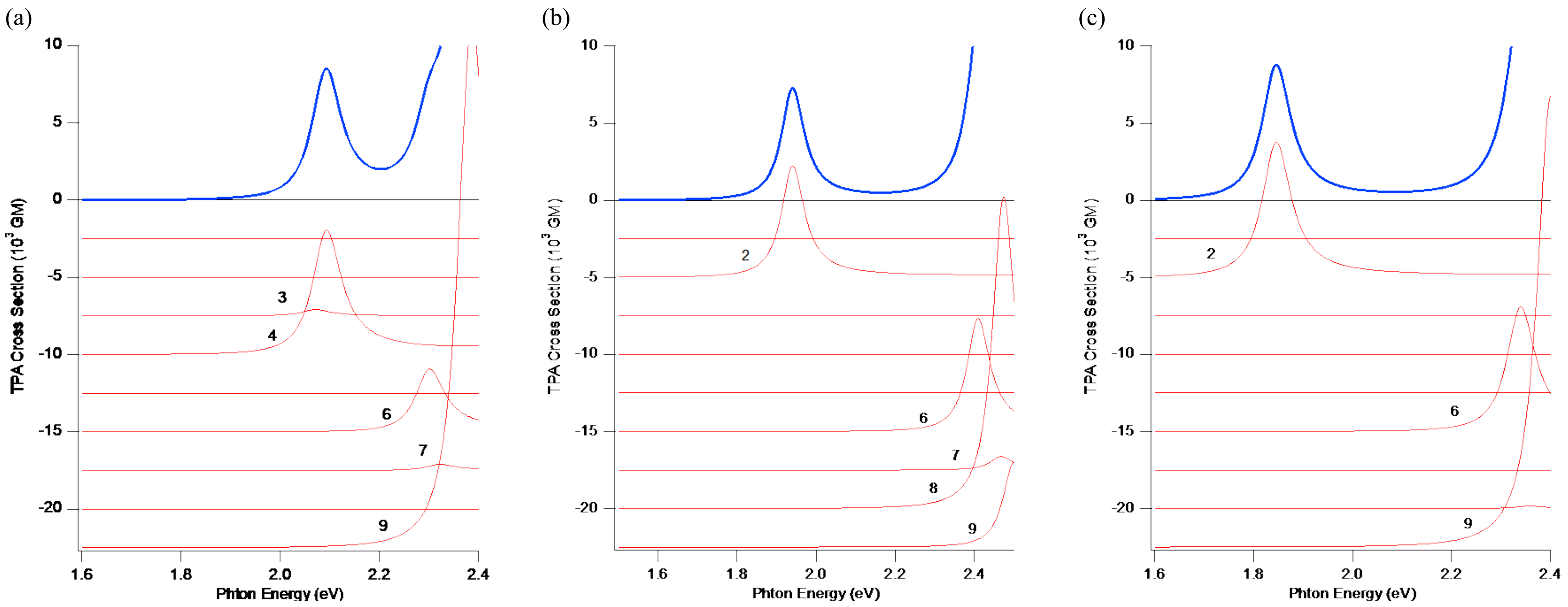

Figure S8. Simulated TPA spectrum (blue line) of **6** (a), **7** (b), and **8** (c) with its decomposed spectrum based by destination excited state (red curves with fill, S_1–S_9 to from top to bottom, sifted for readability). The numbers by the decomposed spectrum are the number of the destination excited states. Calculation level was TDA-CAM-B3LYP/6-31+G(d) with PCM (dichloromethane). For the details of the calculation methods, see Experimental in SI.

4. Quantum chemical calculations

Dye 6

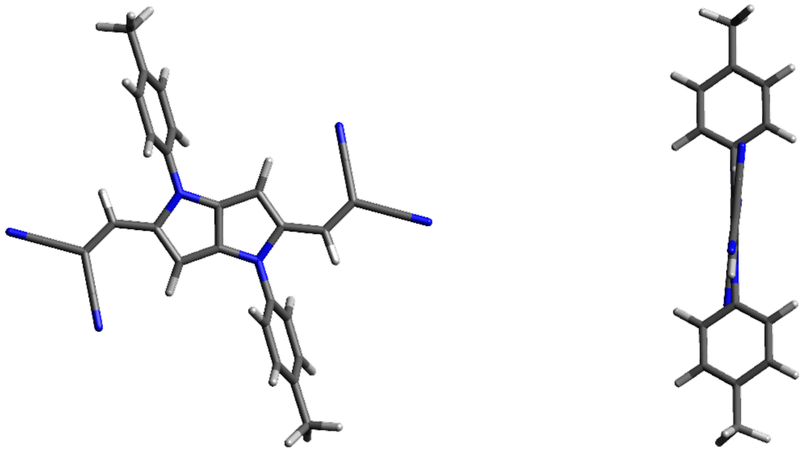

Dye 7

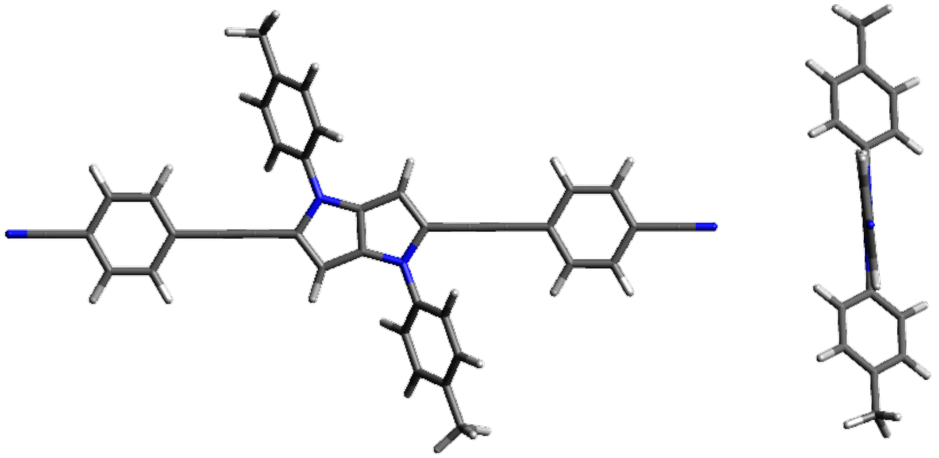

Dye 8

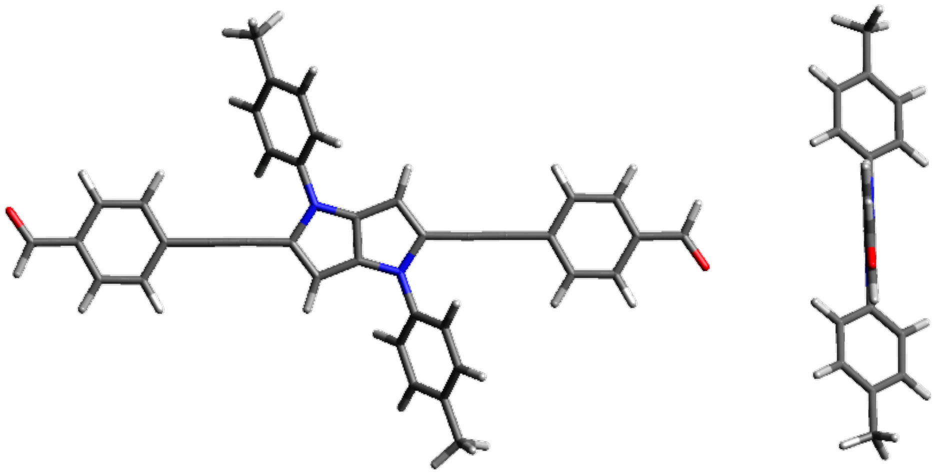

Figure S9. Optimized geometry of dyes **6-8** at the CAM-B3LYP/6-31+G(d) level of theory with the PCM (dichloromethane) used to model solvent effects. The dihedral angle between the *N*-phenyl and pyrrole rings are 69° for **6**, ca.54° for **7** and **8**.

Table S1. Excited states of Dye **6** calculated at the TDA-CAM-B3LYP/6-31+G(d) level of theory with the PCM (dichloromethane) used to model solvent effects.

S1: 2.8792 eV 430.62 nm f=1.8970	
108 → 116	-0.11030
114 → 115	0.68893
S2: 3.3380 eV 371.43 nm f=0.2953	
113 → 115	0.69135
S3: 4.1791 eV 296.68 nm f=0.0000	
112 → 115	0.66383
113 → 116	-0.17912
114 → 116	-0.10907
S4: 4.2651 eV 290.69 nm f=0.0000	
113 → 116	-0.20672
114 → 116	0.64833
S5: 4.4843 eV 276.48 nm f=0.0011	
109 → 115	-0.31874
111 → 115	0.60392
112 → 116	0.12367
S6: 4.6164 eV 268.57 nm f=0.0000	
109 → 116	-0.10120
111 → 116	0.10654
112 → 115	0.19664
113 → 116	0.61430
114 → 116	0.18986
S7: 4.6378 eV 267.33 nm f=0.0000	
110 → 115	0.69317
S8: 4.6472 eV 266.80 nm f=0.0359	
109 → 115	0.61548
110 → 116	0.10302
111 → 115	0.32738
S9: 4.8272 eV 256.84 nm f=0.0000	
108 → 115	0.66831
113 → 116	-0.10101
S10: 5.3785 eV 230.52 nm f=0.0043	
107 → 123	-0.10610
108 → 122	0.22018
108 → 125	0.12681
114 → 118	0.17525
114 → 119	-0.13632
114 → 121	-0.32302
114 → 123	0.46717
114 → 126	0.10938

Table S2. Excited states of Dye **7** calculated at the TDA-CAM-B3LYP/6-31+G(d) level of theory with the PCM (dichloromethane) used to model solvent effects.

S1: 3.1720 eV 390.87 nm f=3.0261	
138 → 142	0.15304
140 → 141	0.67042
S2: 3.9951 eV 310.34 nm f=0.0000	
138 → 141	0.20176
140 → 142	0.64349
S3: 4.1348 eV 299.85 nm f=0.2540	
139 → 141	0.66418
139 → 145	0.15171
S4: 4.8083 eV 257.86 nm f=0.0005	
131 → 141	0.18101
131 → 142	0.19163
132 → 141	0.23372
132 → 142	-0.17598
133 → 143	0.13611
138 → 144	0.25650
140 → 143	0.44901
140 → 145	-0.16767
S5: 4.8096 eV 257.79 nm f=0.0000	
131 → 141	0.23482
131 → 142	0.17649
132 → 141	-0.18168
132 → 142	0.19259
133 → 144	0.15546
138 → 143	0.22925
138 → 145	-0.11748
140 → 144	0.47507
S6: 4.8255 eV 256.93 nm f=0.0000	
137 → 141	0.14928
138 → 141	0.33620
139 → 142	0.51924
139 → 146	0.12265
140 → 142	-0.15750
S7: 4.9552 eV 250.21 nm f=0.0018	
140 → 143	-0.12504
140 → 145	-0.23503
140 → 146	0.35155
140 → 147	0.41604
140 → 149	0.11415
140 → 151	0.13286
140 → 154	-0.13346
S8: 4.9619 eV 249.87 nm f=0.0001	
133 → 142	0.14543
137 → 141	-0.26390
138 → 141	0.43672
139 → 142	-0.28654
140 → 142	-0.12868
140 → 145	-0.12023
140 → 146	-0.11527
140 → 147	0.17698
S9: 5.0125 eV 247.35 nm f=0.0041	
138 → 141	0.15958
140 → 143	0.18083
140 → 145	0.39363
140 → 146	0.33319
140 → 148	0.17253
140 → 161	-0.20307
140 → 169	-0.13234
S10: 5.0432 eV 245.85 nm f=0.0052	
140 → 143	0.17576
140 → 145	0.36368
140 → 146	-0.18126
140 → 147	0.33839
140 → 148	-0.12595
140 → 161	0.22027
140 → 169	0.14176

Table S3. Excited states of Dye **8** calculated at the TDA-CAM-B3LYP/6-31+G(d) level of theory with the PCM (dichloromethane) used to model solvent effects.

S1: 3.0767 eV 402.98 nm f=2.9937	
140 → 144	0.15971
**142 → 143**	**0.66332**
142 → 145	0.12022
S2: 3.8076 eV 325.62 nm f=0.0000	
140 → 143	0.19814
**142 → 144**	**0.64721**
S3: 3.9518 eV 313.74 nm f=0.0001	
**131 → 143**	**0.42367**
**131 → 144**	**0.43333**
131 → 145	-0.22800
131 → 157	-0.15052
132 → 144	0.10265
S4: 3.9519 eV 313.74 nm f=0.0001	
131 → 144	-0.10285
**132 → 143**	**-0.42289**
**132 → 144**	**0.43409**
132 → 145	0.22726
132 → 157	-0.15088
S5: 4.0835 eV 303.62 nm f=0.2236	
141 → 143	0.65670
141 → 145	0.20757
S6: 4.6873 eV 264.51 nm f=0.0000	
139 → 143	-0.13254
140 → 143	-0.30989
141 → 144	0.54938
141 → 148	-0.10892
142 → 144	0.14459
S7: 4.7158 eV 262.91 nm f=0.0155	
133 → 143	0.35796
134 → 144	-0.33183
135 → 143	-0.12958
135 → 147	-0.13727
140 → 146	0.22487
142 → 145	0.20632
142 → 147	0.29187
S8: 4.7230 eV 262.51 nm f=0.0000	
133 → 144	-0.32308
134 → 143	0.38846
135 → 144	0.10365
135 → 146	0.14534
140 → 147	-0.20349
142 → 146	-0.34415
S9: 4.8323 eV 256.57 nm f=0.0000	
135 → 144	-0.16397
139 → 143	-0.21107
140 → 143	0.50692
141 → 144	0.30923
142 → 144	-0.16511
S10: 4.8648 eV 254.86 nm f=0.0291	
142 → 145	0.57927
142 → 147	-0.26311
142 → 149	-0.13619
142 → 152	-0.11234

Table S4. Summary of the calculated parameters at the TDA-CAM-B3LYP/6-31+G(d) level of theory for the first few TPA transitions. Transition path is f←i←0, where destination state (f), intermediate state (i), and the ground state (0). Absolute values of transition dipole moments ($|\boldsymbol{\mu}_{fi}|$ and $|\boldsymbol{\mu}_{i0}|$), transition energies (E_f and E_i), detuning energy ($\Delta E_{det} = E_i - E_f/2$), the angle ($\theta$) between the transition dipole moments $\boldsymbol{\mu}_{fi}$ and $\boldsymbol{\mu}_{i0}$. The three-state model was used to calculate the relative magnitude of the component of TPA cross section via the f←i←0 path ($\sigma_{TPA,rel} = F_{TDM}\, F_{det}\, F_{ang}$), where TPA transition dipole moments factor ($F_{TDM} = |\boldsymbol{\mu}_{fi}|^2 |\boldsymbol{\mu}_{i0}|^2$), detuning-energy factor ($F_{det} = \left(\frac{E_f/2}{E_i - E_f/2}\right)^2 = [\Delta E_{det}/(E_f/2)]^{-2}$), angle factor ($F_{ang} = 1 + 2\cos^2\theta$). See ref. S8 for the detailed definitions.

cmpd	f←i←0	$\lvert\boldsymbol{\mu}_{fi}\rvert$ / D	$\lvert\boldsymbol{\mu}_{i0}\rvert$ /D	E_f / eV	E_i / eV	ΔE_{det} / eV	θ / °	F_{TDM} / D^4	F_{det}	F_{ang}	$\sigma_{TPA,rel}$
Dye 6											
	4←1←0	11.1	13.9	4.18	2.68	0.59	0.4	23704	12.5	3.00	295352
	6←1←0	3.6	13.9	4.60	2.68	0.38	11.2	2503	35.6	2.92	89207
	9←1←0	10.0	13.9	4.76	2.68	0.30	4.1	19571	62.7	2.99	1227102
Dye 7											
	2←1←0	17.2	16.3	3.88	3.06	1.12	178.3	78715	3.0	3.00	235326
	6←1←0	8.7	16.3	4.81	3.06	0.66	0.5	20022	13.5	3.00	270297
	8←1←0	11.9	16.3	4.94	3.06	0.59	3.2	37418	17.6	2.99	658557
Dye 8											
	2←1←0	19.7	16.5	3.69	2.97	1.13	179	105983	2.7	3.00	285094
	6←1←0	8.4	16.5	4.67	2.97	0.64	178	19006	13.7	3.00	260382
	9←1←0	14.3	16.5	4.80	2.97	0.57	3.8	55561	17.7	2.99	982318

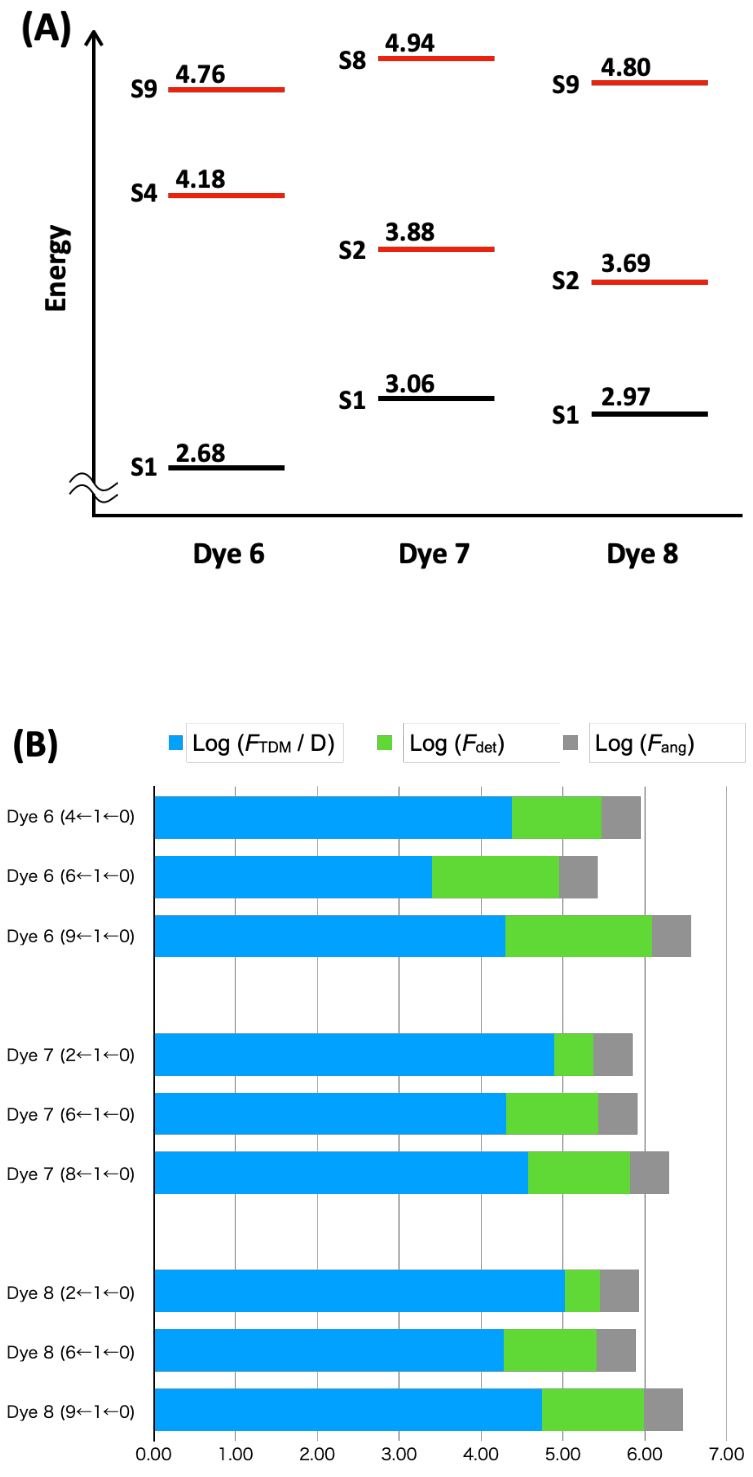

Figure S10. (A) Energy diagram of the calculated excited states of the lowest OPA state (black line) and two major TPA sates (red line). The numbers are the energy from the ground state. (B) Graphical representation of the factors consisting of TPA cross sections ($\sigma_{\mathrm{TPA,rel}} = F_{\mathrm{TDM}}\, F_{\mathrm{det}}\, F_{\mathrm{ang}}$) in log scale, i.e., $\log(\sigma_{\mathrm{TPA,rel}}) = \log(F_{\mathrm{TDM}}) + \log(F_{\mathrm{det}}) + \log(F_{\mathrm{ang}})$, where transition dipole moments factor (F_{TDM}), detuning-energy factor (F_{det}), angle factor (F_{ang}), see the caption of Table S4. All data from Table S4. Unity in the horizontal scale means 10-folds difference.

5. Spectroscopic properties

Table S5. Spectroscopic properties of dyes **6**–**8** in different solvents.

Compound	Solvent	λ_{abs}^{max} [nm]	ε_{max} [$M^{-1}cm^{-1}$]	λ_{em}^{max} [nm]	Stokes shift [cm^{-1}]	φ_{fl}	$2\lambda_{abs}^{max}$ [nm]	λ_{TPA}^{max} [nm]	σ_{TPA}^{max} [GM]	σ_{TPA}^{max} φ_{fl} [GM]
6	CHX	501	92200	509	314	0.009[a]				
		472	59700							
	PhMe	512	62600	536	875	0.012[a]				
		483	50500							
	THF	507	66200	532	927	0.009[a]				
		480	55000							
	DCM	516	73000	540	861	0.013[a]	1032	648	505±95	6.6
		481	56000				962	577	(1340±NA)	17
	ACN	505	59100	540	1280	0.007[a]				
		481	52100							
7	CHX	443	116800	451	400	0.51[b]				
		419	84800							
	PhMe	442	88800	466	1170	0.41[b]				
		419	78800							
	THF	439	81500	478	1860	0.15[b]				
	DCM	441	81700	486	2100	0.06[b]	882	721	1050±190	63
	ACN	430	80100	504	3420	0.01[b]				
8	CHX	459	92200	469	564	0.53[b]				
		435	68600							
	PhMe	458	71200	493	1550	0.46[b]				
	THF	452	75900	526	3110	0.10[b]				
	DCM	455	71000	566	4310	0.02[b]	910	770	825±160	17
	ACN	442	66200	581	5410	0.002[b]				

[a]Determined with fluorescein in 0.1M NaOH as a standard. [b]Determined with coumarin 153 in ethanol as a standard. CHX – cyclohexane, PhMe – toluene, THF – tetrahydrofuran, DCM – dichloromethane, ACN – acetonitrile.

6. Absorption and emission spectra

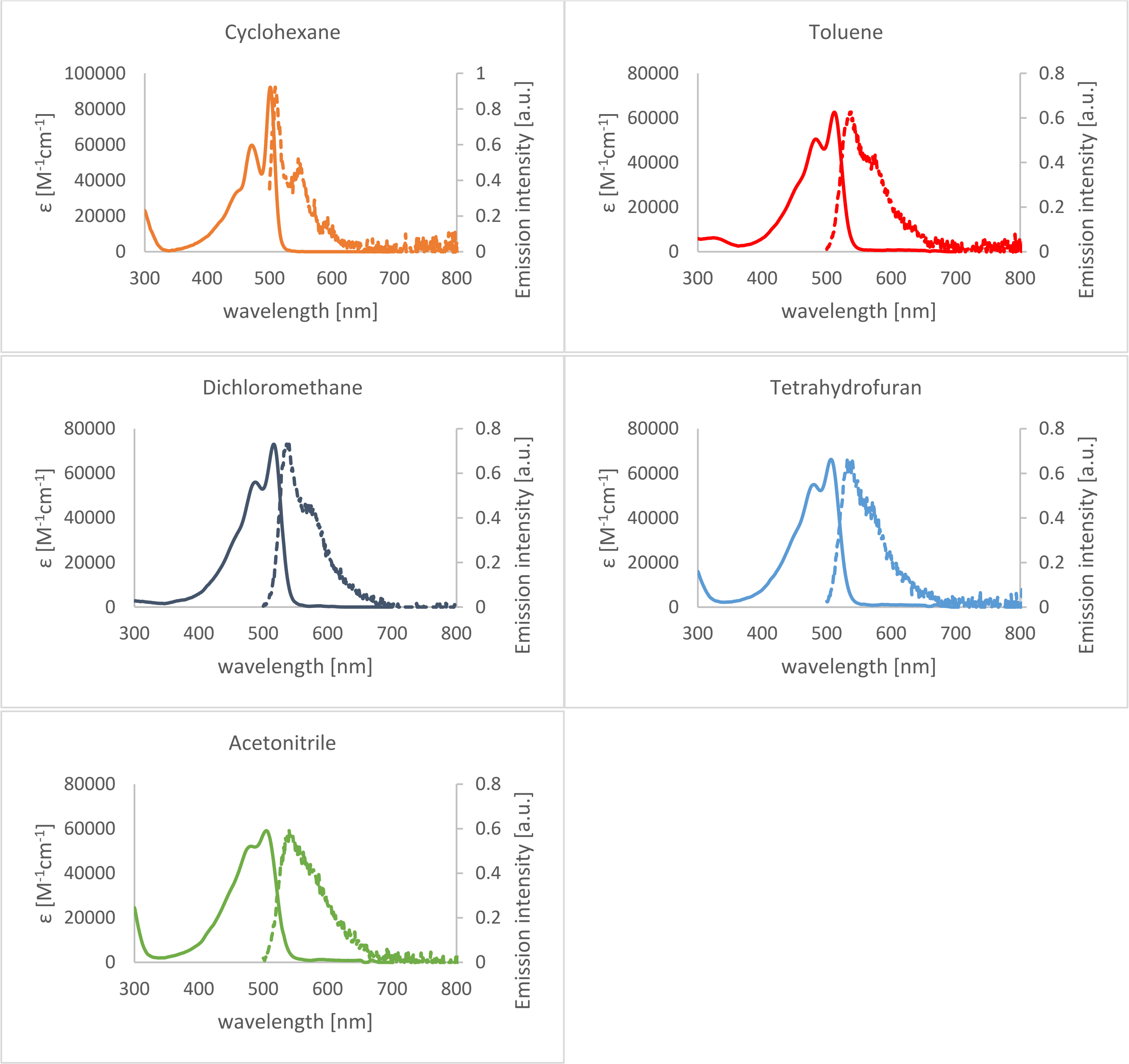

Figure S11. Absorption (solid lines) and normalized emission (dashed lines) spectra of compound **6** measured in different solvents.

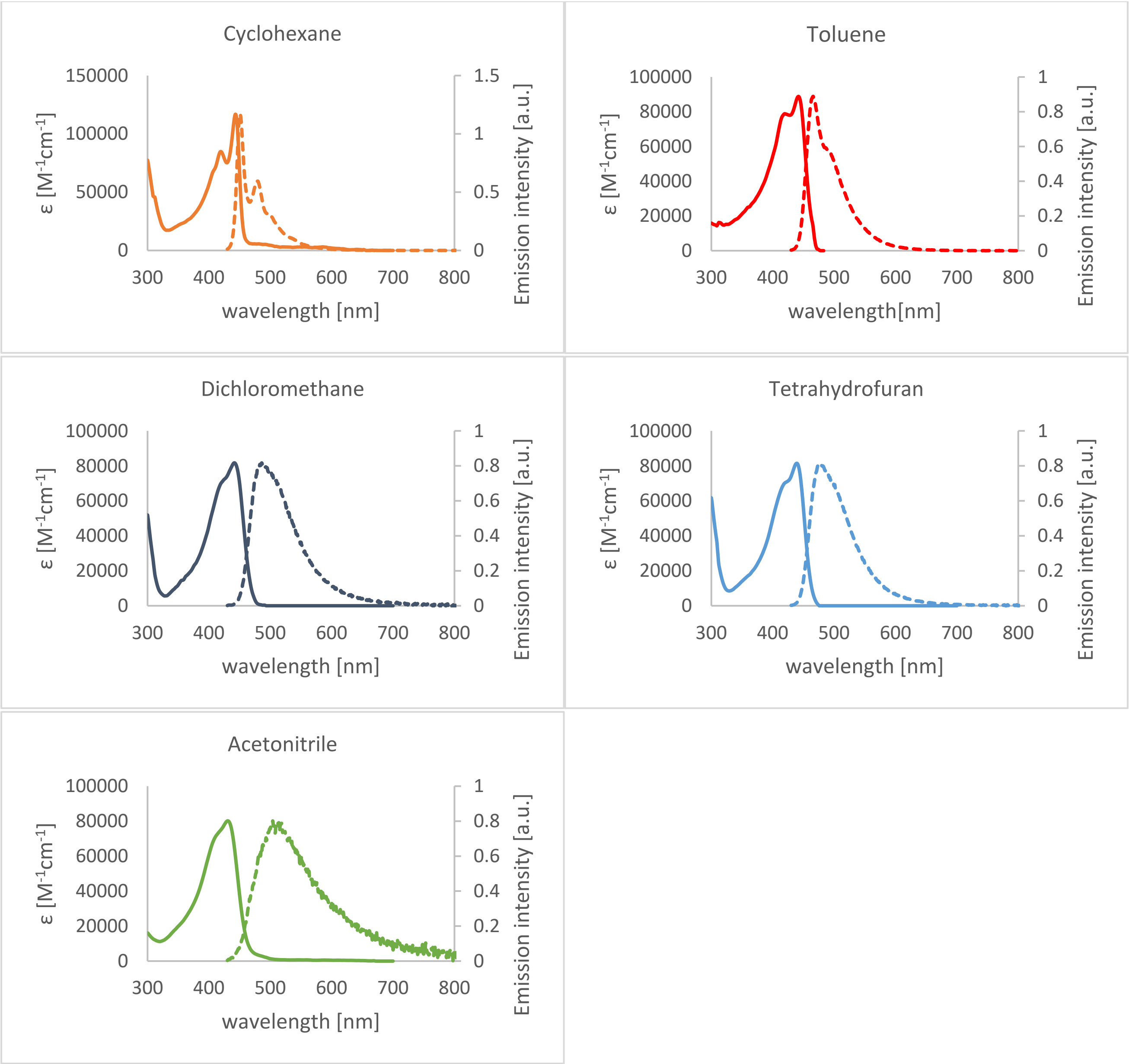

Figure S12. Absorption (solid lines) and normalized emission (dashed lines) spectra of compound **7** measured in different solvents.

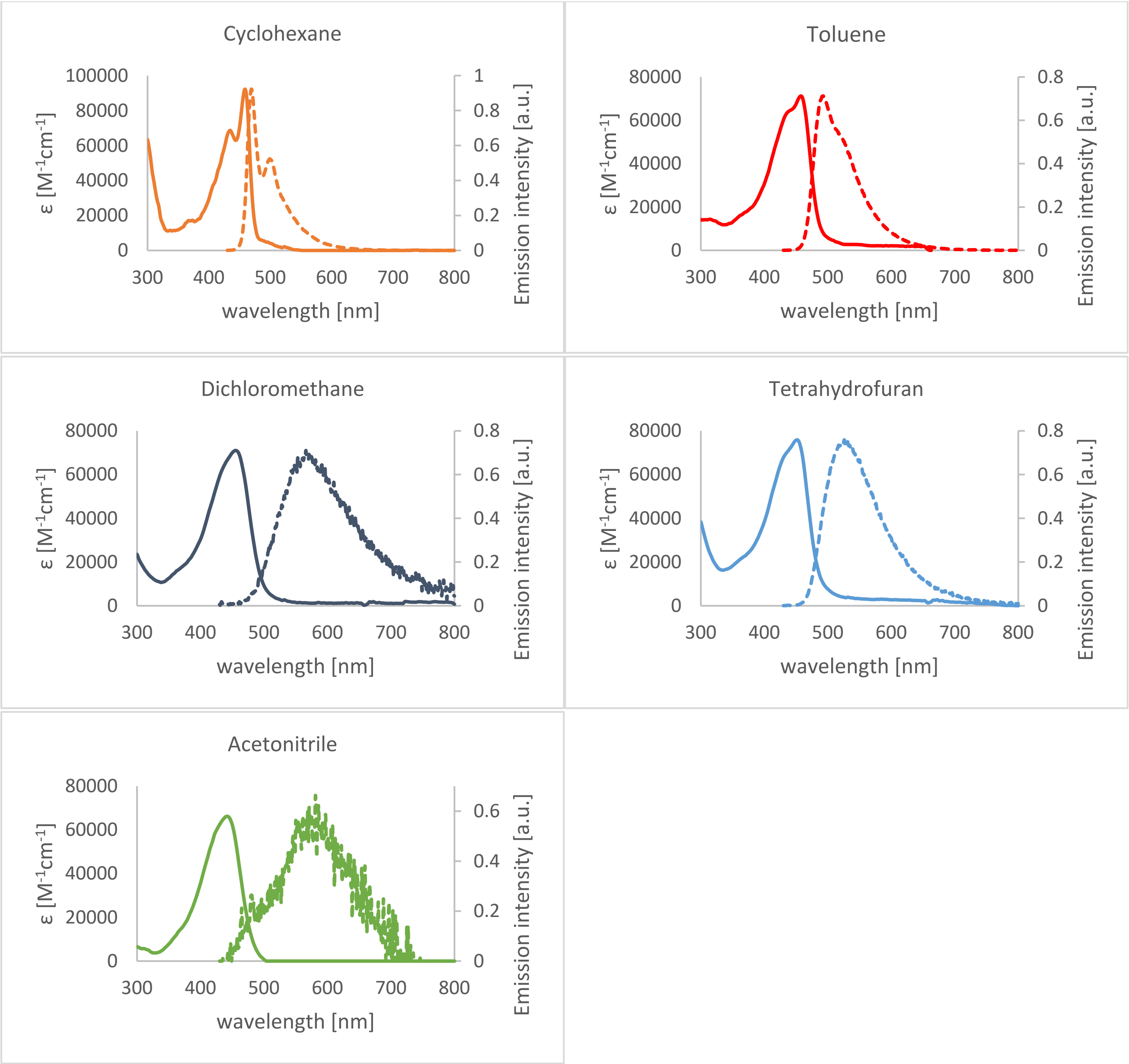

Figure S13. Absorption (solid lines) and normalized emission (dashed lines) spectra of compound **8** measured in different solvents.

8. ^{1}H and ^{13}C NMR spectra

^{1}H NMR (500 MHz, $CDCl_3$) of **4**

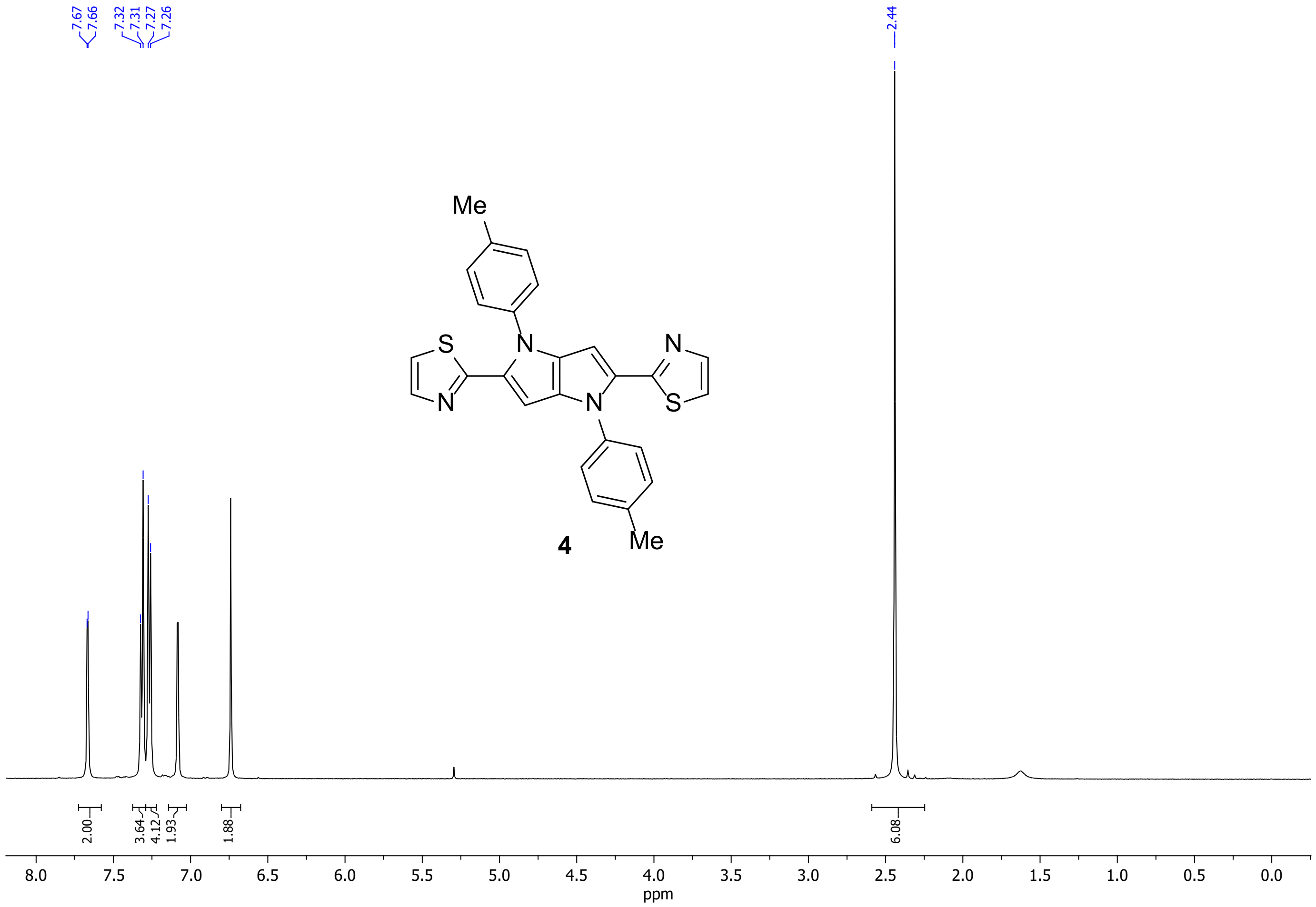

^{1}H NMR (500 MHz, $CDCl_3$) of **5**

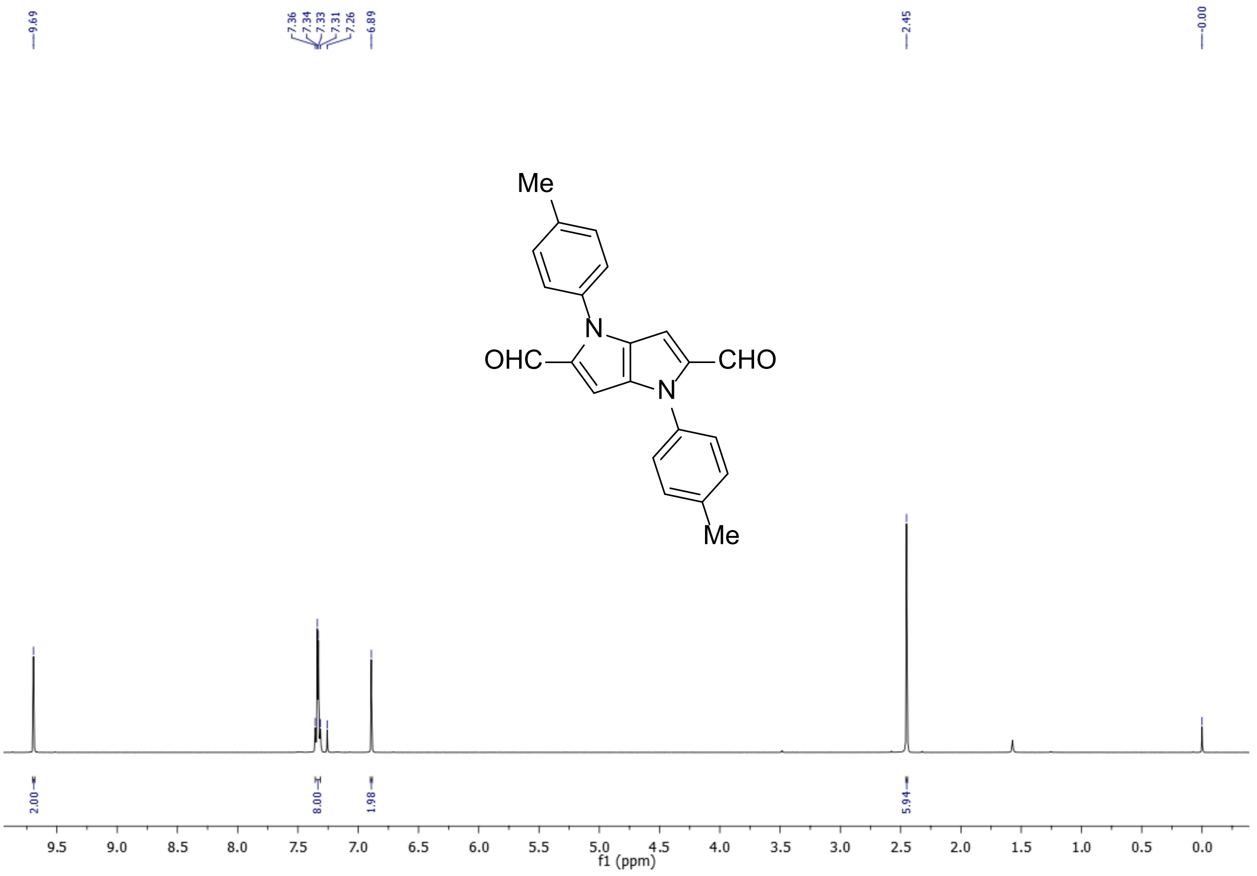

$^{13}C\{^{1}H\}$ NMR (126 MHz, $CDCl_3$) of **5**

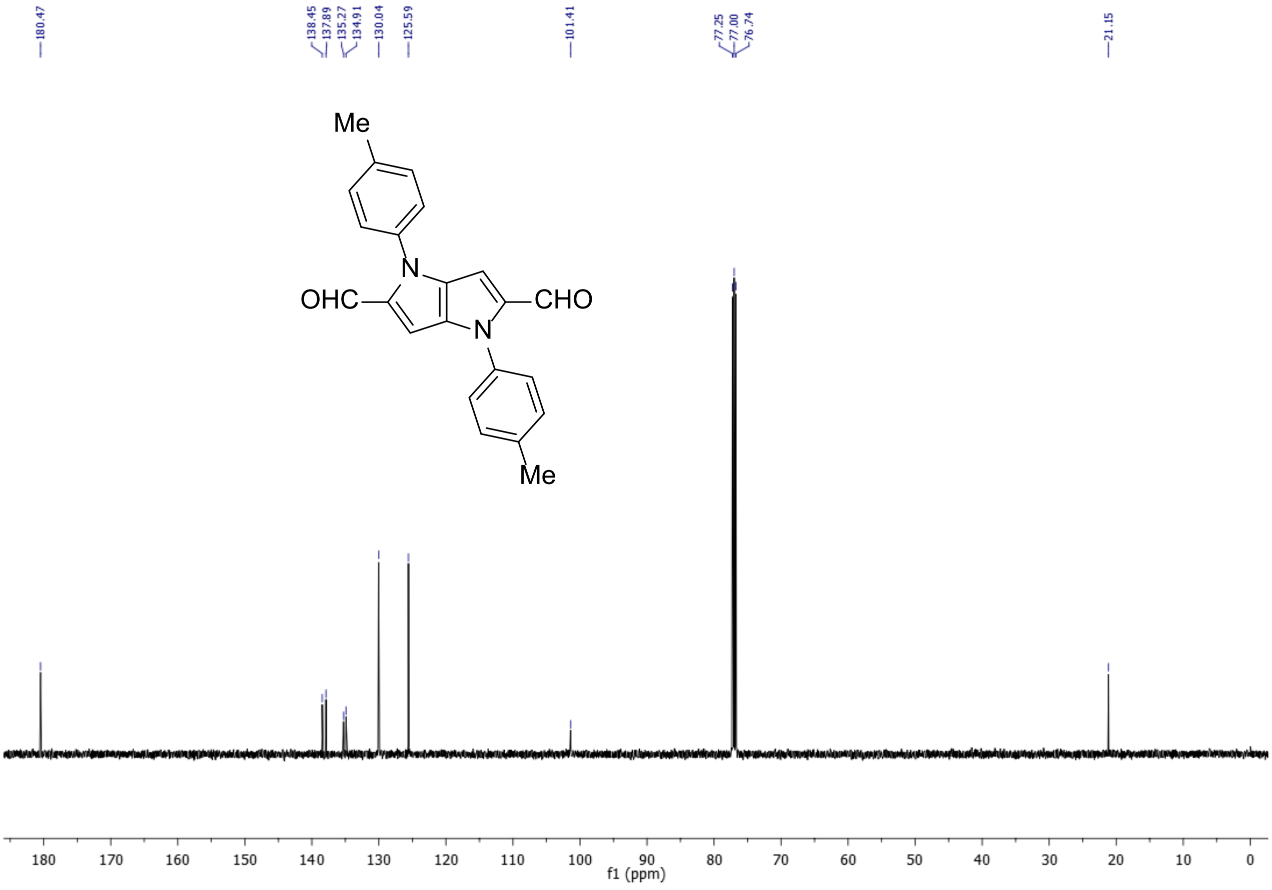

^1H NMR (500 MHz, $CDCl_3$) of **6**

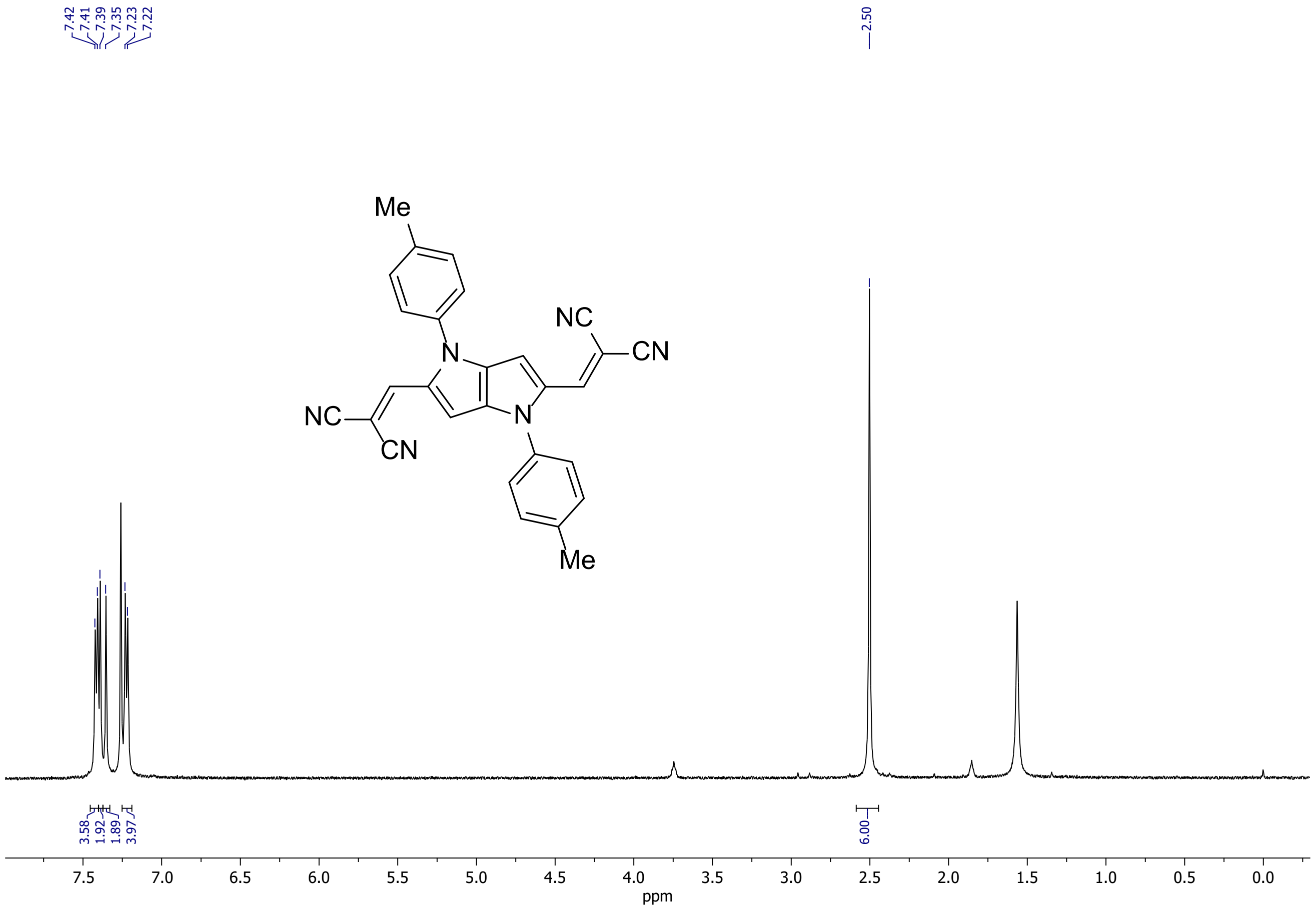

$^{13}C\{^1H\}$ NMR (126 MHz, $CDCl_3$) of **6**

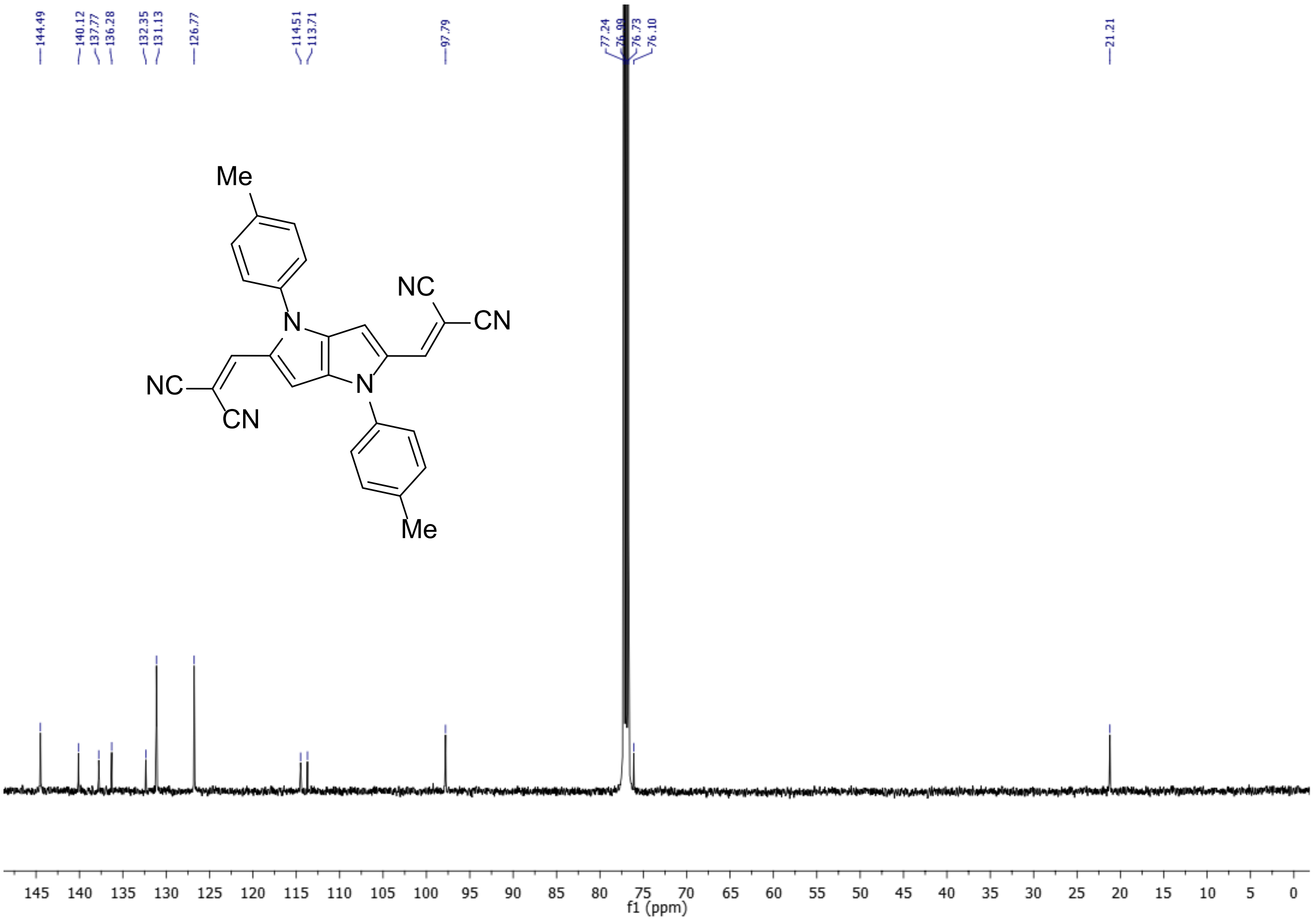

^{1}H NMR (500 MHz, $CDCl_3$) of **7**

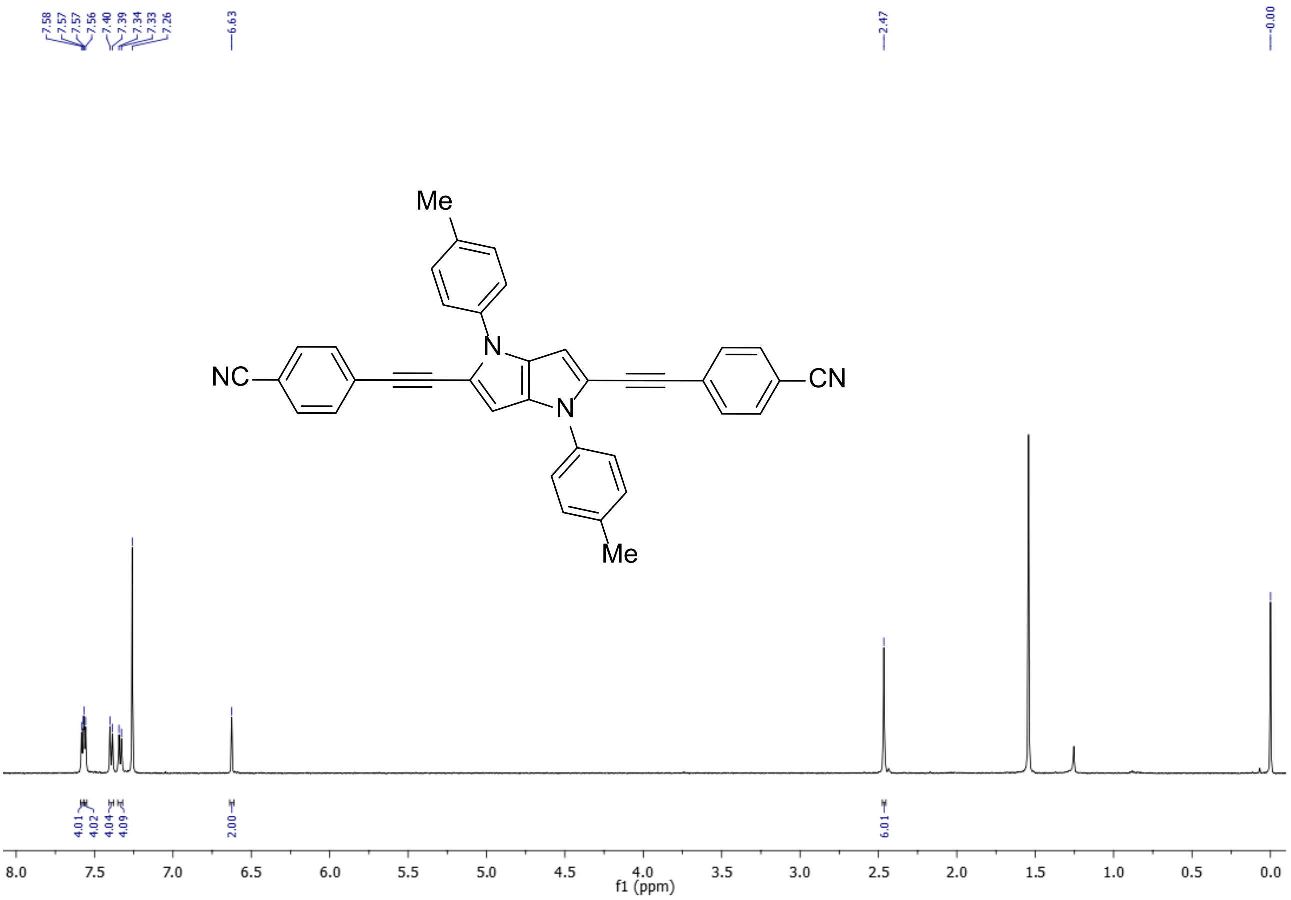

$^{13}C\{^{1}H\}$ NMR (126 MHz, $CDCl_3$) of **7**

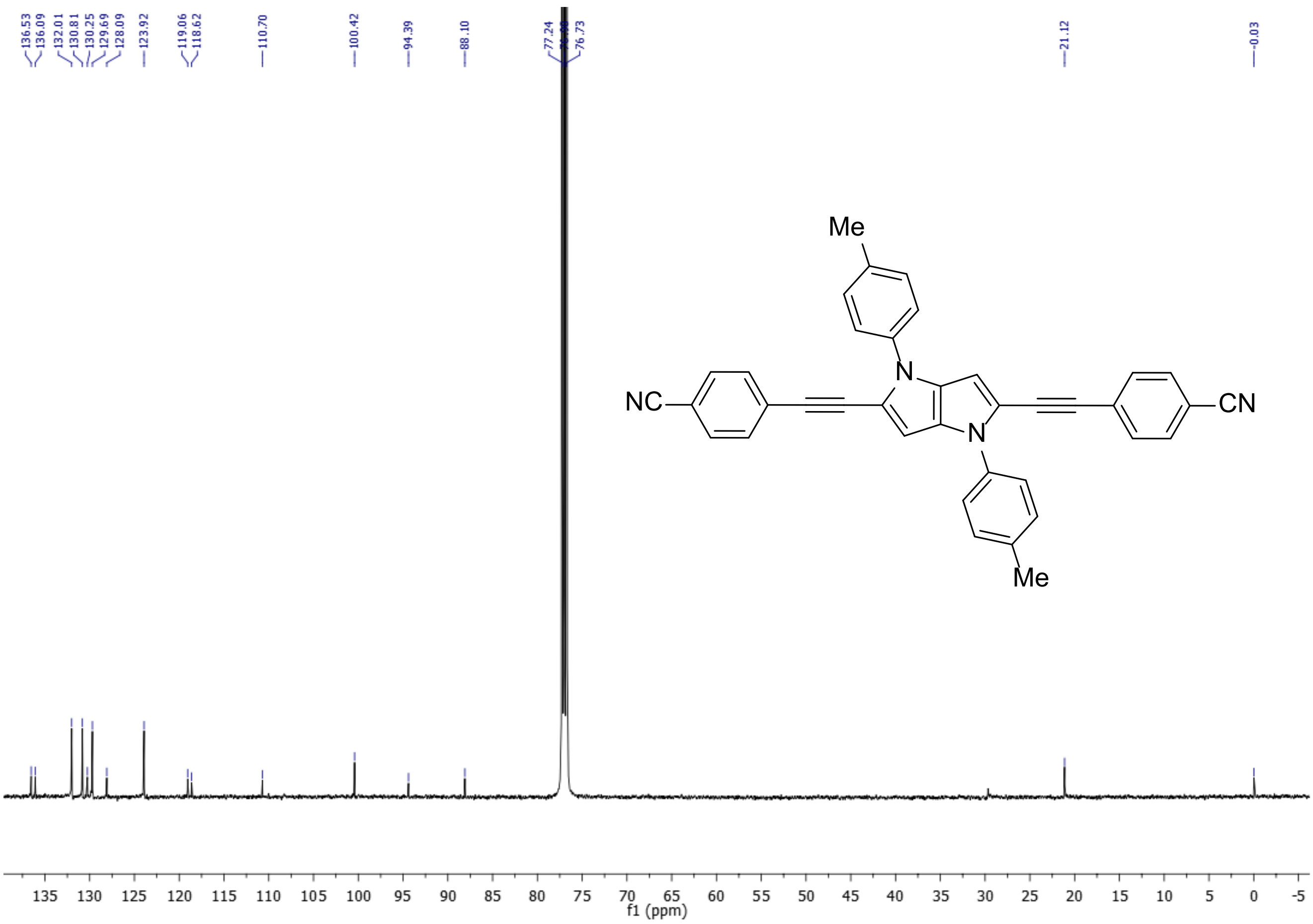

^{1}H NMR (500 MHz, $CDCl_3$) of **8**

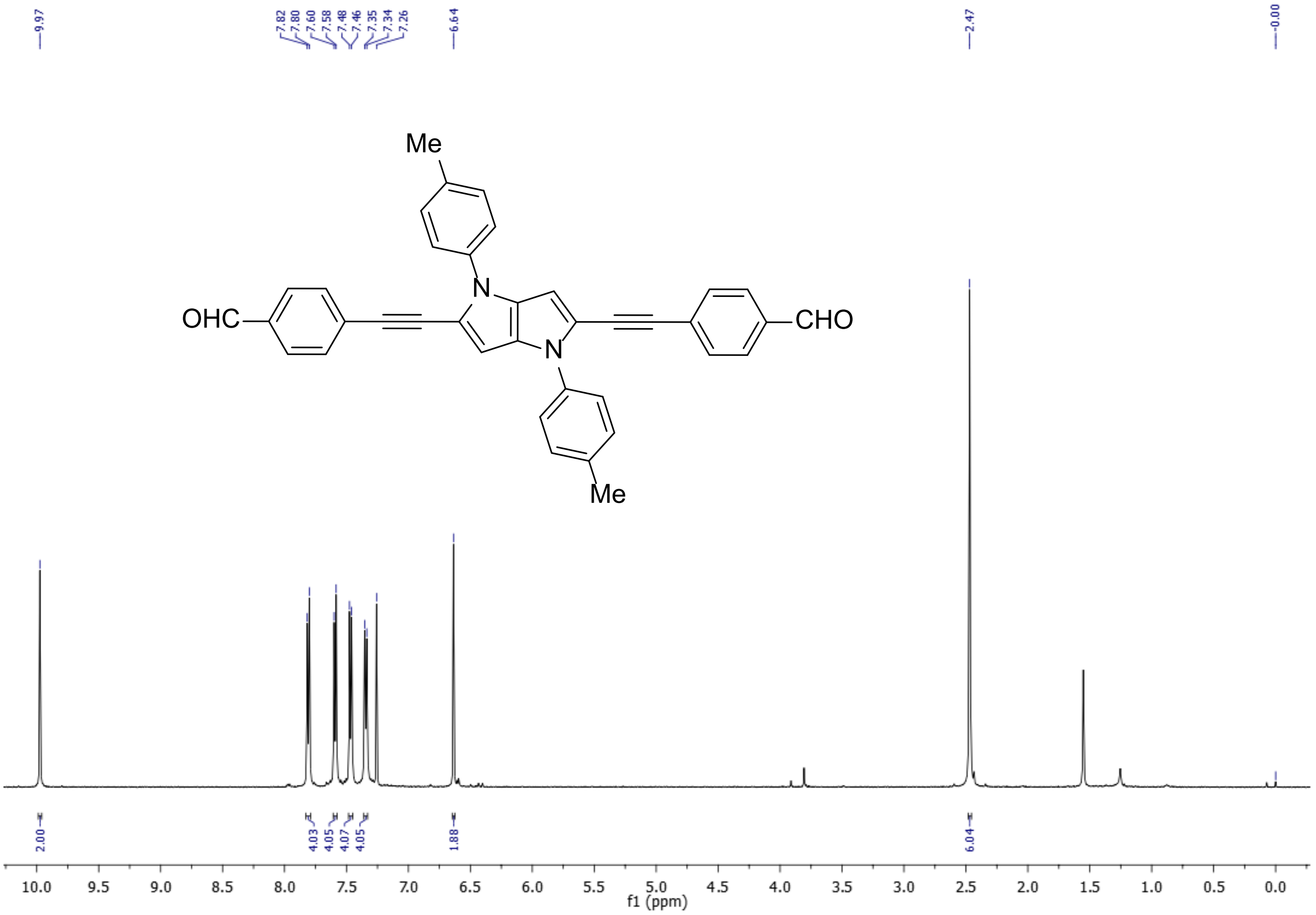

^{13}C{^{1}H} NMR (126 MHz, $CDCl_3$) of **8**

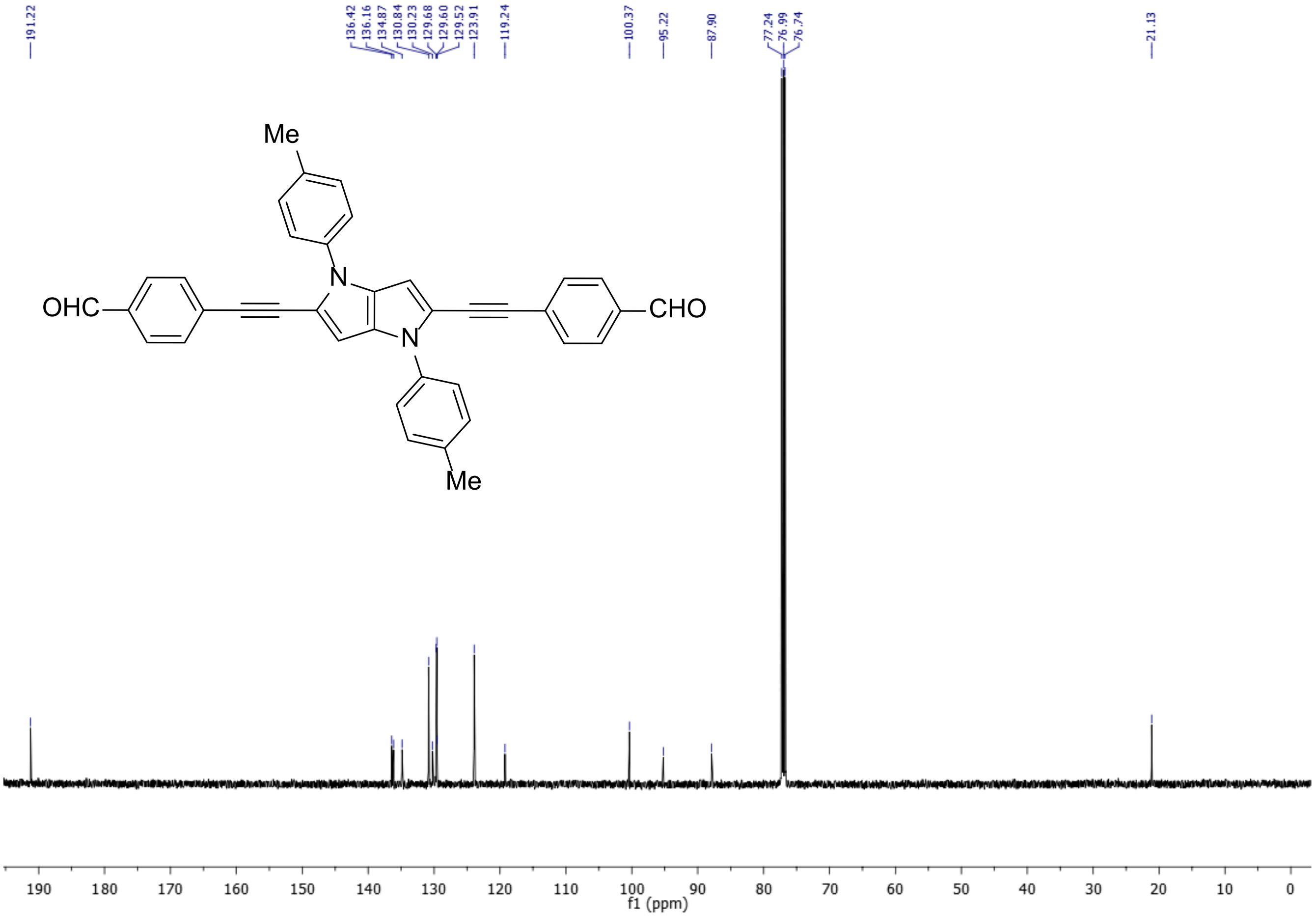

9. References

(1) M. Sheik-Bahae, A. A. Said, T. H. Wei, D. J. Hagan, E. W. van Stryland, *IEEE J. Quantum Electron.* **1990**, 26, 760.

(2) K. Kamada, K. Matsunaga, A. Yoshino, K. Ohta, *J. Opt. Soc. Am. B* **2003**, 20, 529.

(3) K. Kamada, Y. Iwase, K. Sakai, K. Kondo, K. Ohta, *J. Phys. Chem. C* **2009**, 113, 11469.

(4) K. Górski, I. Deperasińska, G. V. Baryshnikov, S. Ozaki, K. Kamada, H. Ågren, D. T. Gryko, *Org. Lett.* **2021**, 23, 6770.

(5) K. Kamada, C. Hara, K. Ogawa, K. Ohta, Y. Kobuke, *Chem. Comm.* **2012**, 48, 7988.

(6) S. Hirata, M. Head-Gordon, *Chem. Phys. Lett.* **1999**, 314, 291.

(7) Gaussian 16, Revision C.01, M. J. Frisch, G. W. Trucks, H. B. Schlegel, G. E. Scuseria, M. A. Robb, J. R. Cheeseman, G. Scalmani, V. Barone, G. A. Petersson, H. Nakatsuji, X. Li, M. Caricato, A. V. Marenich, J. Bloino, B. G. Janesko, R. Gomperts, B. Mennucci, H. P. Hratchian, J. V. Ortiz, A. F. Izmaylov, J. L. Sonnenberg, D. Williams-Young, F. Ding, F. Lipparini, F. Egidi, J. Goings, B. Peng, A. Petrone, T. Henderson, D. Ranasinghe, V. G. Zakrzewski, J. Gao, N. Rega, G. Zheng, W. Liang, M. Hada, M. Ehara, K. Toyota, R. Fukuda, J. Hasegawa, M. Ishida, T. Nakajima, Y. Honda, O. Kitao, H. Nakai, T. Vreven, K. Throssell, J. A. Montgomery, Jr., J. E. Peralta, F. Ogliaro, M. J. Bearpark, J. J. Heyd, E. N. Brothers, K. N. Kudin, V. N. Staroverov, T. A. Keith, R. Kobayashi, J. Normand, K. Raghavachari, A. P. Rendell, J. C. Burant, S. S. Iyengar, J. Tomasi, M. Cossi, J. M. Millam, M. Klene, C. Adamo, R. Cammi, J. W. Ochterski, R. L. Martin, K. Morokuma, O. Farkas, J. B. Foresman, and D. J. Fox, Gaussian, Inc., Wallingford CT, 2019.

(8) J.B. Foresman, A. Frisch, “Exploring Chemistry with Electronic Structure Methods”, 2nd Ed. (1993) Gaussian Inc., Pittsburgh.

(9) K. Ohta, S. Yamada, K. Kamada, A. D. Slepkov, F. A. Hegmann, R. R. Tykwinski, L. D. Shirtcliff, M. M. Haley, P. Sałek, F. Gel’mukhanov, H. Ågren, *J. Phys. Chem. A* **2011**, 115, 105.

(10) Tasior, M.; Koszarna, B.; Young, D. C.; Bernard, B.; Jacquemin, D.; Gryko, D.; Gryko, D. T. *Org. Chem. Front.*, **2019**, *6*, 2939-2948.
